# Structure-based discovery and definition of RiPP recognition elements

**DOI:** 10.1128/msystems.01252-25

**Published:** 2025-11-18

**Authors:** Miriam H. Bregman, Dillon P. Cogan, Kyle E. Shelton, Andrew J. Rice, Shravan R. Dommaraju, Satish K. Nair, Douglas A. Mitchell

**Affiliations:** 1 Department of Chemistry, University of Illinois at Urbana-Champaign 14589, Urbana, Illinois, USA; 2 Department of Biochemistry, University of Illinois at Urbana-Champaign 14589, Urbana, Illinois, USA; 3 Department of Biochemistry, School of Medicine – Basic Sciences, Vanderbilt University 5718, Nashville, Tennessee, USA; 4 Carl R. Woese Institute for Genomic Biology, University of Illinois at Urbana-Champaign 14589, Urbana, Illinois, USA; 5 Center for Biophysics and Quantitative Biology, University of Illinois at Urbana-Champaign 14589, Urbana, Illinois, USA; 6 Department of Chemistry, Vanderbilt University 5718, Nashville, Tennessee, USA; Universiteit Leiden, Leiden, the Netherlands

**Keywords:** genome mining, RiPPs, ribosomal peptides, natural products, molecular recognition, protein:peptide interactions

## Abstract

Ribosomally synthesized and post-translationally modified peptides (RiPPs) are a large class of natural products with wide-ranging structural and functional diversity. Central to many RiPP biosynthetic pathways is the RiPP Recognition Element (RRE), a structurally conserved peptide-binding domain that enables class-independent genome mining. Bioinformatic tools, such as RRE-Finder, have leveraged this domain to identify novel RiPPs, but accuracy has been limited by high false-positive rates. To improve accuracy, we assessed whether structure-based searching of the AlphaFold database with the rapid tertiary structure alignment tool Foldseek could reduce false-positive rates and identify previously unretrievable, sequence-divergent RREs. We used these divergent RREs to build 11 new Foldseek-derived Hidden Markov Models (HMMs) and refined existing models through improved seed alignments, domain excision, bit score thresholds, and Pfam filters. Improved precision mode HMMs retrieved nearly twice as many RREs from the UniProt database as the original models, including novel domain fusions. In total, the updated workflow identified >90,000 high-confidence RREs. To further characterize these RREs and assess their functional relevance, we used a combined bioinformatic and AlphaFold 3 approach to predict over 8,000 RRE–peptide complexes. This enabled the mapping of 13 distinct recognition sequences across known RiPP classes. We further validated the ability of AlphaFold to predict precursor peptide interactions with their cognate RRE domains through binding assays to streamline recognition sequence and putative substrate identification. Together, these improvements enhance the accuracy and scope of RRE-Finder, improving access to previously hidden RRE-dependent biosynthetic pathways.

**IMPORTANCE:**

Genome mining relies heavily on sequence similarity searches, which severely limit the discovery potential for sequence-divergent proteins. To mitigate this challenge for RRE domain discovery, we employed structure-based alignments to predict sequence-divergent RREs using Foldseek. The newly identified RRE domains were then used to build new HMMs for use by RRE-Finder. This process identified 5,000 previously unidentified but high-confidence RRE domains. Representatives in this sequence-divergent group retain the canonical RRE fold but display new domain fusions, offering additional bioinformatic handles for genome mining. In parallel, AlphaFold 3 modeling of RRE-precursor peptide interactions enabled the identification of 13 distinct recognition sequence motifs, spanning many RiPP biosynthetic pathways. These approaches have significantly expanded the RRE-dependent RiPP biosynthetic landscape.

## INTRODUCTION

Ribosomally synthesized and post-translationally modified peptides (RiPPs) constitute a diverse class of natural products with therapeutic and bioengineering potential (1–4). RiPPs are biosynthesized from genetically encoded precursor peptides that are enzymatically modified to form the mature product. RiPP classes are typically defined by the installed modification(s), and bioinformatic discovery of novel RiPPs has largely relied on sequence homology of modifying enzymes. Although some enzymes appear in several distinct RiPP classes (e.g., YcaO [PF02624] and radical SAM proteins [PF04055]), a major challenge in discovering novel RiPP modifications is the absence of widely conserved, class-independent biosynthetic markers (5–8). One such conserved domain is the RiPP recognition element (RRE), a structurally conserved but sequence-divergent domain that binds to the leader (or follower) region of the precursor peptide through contacts with a well-conserved recognition sequence (9).

For this work, we strictly define RREs by a specific N-to-C order of secondary structural elements that compose the winged helix-turn-helix secondary structure: βββ-ααα. Although other functionally related domains exist within RiPP biosynthesis—such as the MbnC C-terminal domain (UniProt: A0A1I4IFH0), which has a reversed order of secondary structural elements (ααα-βββ); PlpY (NCBI: WP_019503879.1), which is too small to adopt the proper RRE fold; and the borosin binding domain of SonA (UniProt: Q8EGW2)—these are not considered RRE domains using the strict structural definition here (Fig.S1) (10–13). Notably, the tertiary structure of RRE domains is retained across diverse RiPP classes despite low sequence identity (14–18). This makes the RRE one of the few reliable class-independent handles for RiPP genome mining (1). RREs are currently found in 21/49 of characterized prokaryotic RiPP classes, where they primarily mediate substrate recognition (14, 19, 20). We previously developed RRE-Finder, a class-independent genome mining tool built with custom HMMs derived from known RREs (precision mode) and broader profiles supported by a truncated HHpred pipeline (exploratory mode) (21, 22).

Although RRE-Finder has successfully enabled the discovery of new divergent RiPP classes, its dependence on previously identified RiPPs and sequence- or profile-based similarity can limit its ability to identify highly divergent RREs (6, 7). This is especially problematic for transcription factors, which often share similar winged-helix folds and contribute disproportionately as false positives (23, 24). The AlphaFold database, which contains over 214 million predicted structures, enables structure-guided mining at a previously inaccessible scale (25, 26). This abundance of structural models opens opportunities for identifying conserved domains like the RRE based on structural conservation rather than sequence homology. Structure-based methods, although historically computationally intensive, offer the potential to annotate distantly related proteins and reveal functional relationships by directly comparing protein folds (27–30).

In this work, we assessed Foldseek, a rapid structural alignment tool, to search for the conserved RRE fold across the AlphaFold protein structure database (31). This approach recovered ~5,000 previously undetected high-confidence RREs, many of which are sequence-divergent and fused to uncharacterized protein domains that are not yet associated with RiPP biosynthesis. These proteins were used to generate new Foldseek-derived HMMs, expanding precision mode and improving detection of structurally conserved, sequence-divergent RREs. Next, we employed AlphaFold 3 to model RRE–peptide recognition sequence complexes, identifying 13 distinct motifs, several of which had not been previously identified within known RiPP biosynthetic gene clusters (BGCs) (32). We determined the 1.23 Å resolution crystal structure for a pyritide (thiopeptide) RRE bound to its cognate leader peptide and carried out *in vitro* characterization of RRE binding for two RiPP pathways. Using the information gleaned about binding interactions, we identified a new recognition sequence. These predictions can help streamline precursor peptide identification in systems lacking canonical sequence motifs. Together, these structure-based strategies expand the RRE-Finder framework and demonstrate how structural genomics can enhance genome mining of cryptic RiPP pathways and other small-molecule biosynthetic systems.

## RESULTS AND DISCUSSION

### Foldseek guides the detection of previously unidentified RREs

To assess whether structure-based mining could recover RREs beyond the scope of existing sequence-based tools, we first tested Foldseek’s ability to retrieve canonical examples, such as PqqD (UniProt: Q8P6M8, Fig. S2) [33]. A Foldseek search with default metrics retrieved 6,672 proteins, of which 6,158 remained after grouping at 40% sequence identity, indicating substantial sequence diversity in the data set. The retrieved proteins were filtered to remove those too small to adopt an RRE fold (<65 amino acids) as well as those belonging to any of 196 Pfam families identified as likely false positives because they lack a structurally predicted RRE fold (see Pfam filtering list) (34, 35). AlphaFold 3-predicted structures were manually assessed to determine a recommended bitscore threshold of 40; ~87% of the data set was predicted to contain an RRE fold. Data analysis showed no obvious correlations between alignment length and false-positive rate. Many false positives were proteins in the expected size range and contained partial structural features, such as a helical bundle, but lacked the fully intact RRE domain. This highlights the difficulty in classifying RREs from Foldseek results alone, particularly for small domains that share common secondary structure motifs with other proteins.

Notably, 50% of total proteins retrieved were annotated as PF05402 (note: this Pfam contains PqqD and many other RRE domains unrelated to pyrroloquinoline quinone, i.e., PQQ, biosynthesis). This represented only ~18% of PF05402-annotated proteins in the AlphaFold database, highlighting the limitation of using single-query input for comprehensive retrieval. In contrast, 26% of retrieved proteins lacked any Pfam annotation, indicating a wide diversity of bioinformatically uncategorized proteins. Manual inspection of AlphaFold 3-predicted structures confirmed the presence of high-confidence putative RREs.

To expand sequence coverage, we ran an earlier version of RRE-Finder’s precision mode, excised RREs using a custom script, and constructed a Sequence Similarity Network (SSN) of the excised RREs (see RRE excision script) (36). Next, a randomly selected representative from each of the 16 largest RRE groups was used as input for Foldseek searches. This procedure retrieved 137,726 proteins using a bitscore cutoff of 40.

After applying the previously described size and Pfam filters, the data set was refined to 99,650 proteins, of which only 4,977 were annotated as members of PF05402. This is another substantial underrepresentation, as the AlphaFold database contains ~19,000 proteins in PF05402 that share the same fold and exhibit high sequence similarity; however, most were not retrieved even when multiple sequence-diverse queries were used.

**Fig 1:**
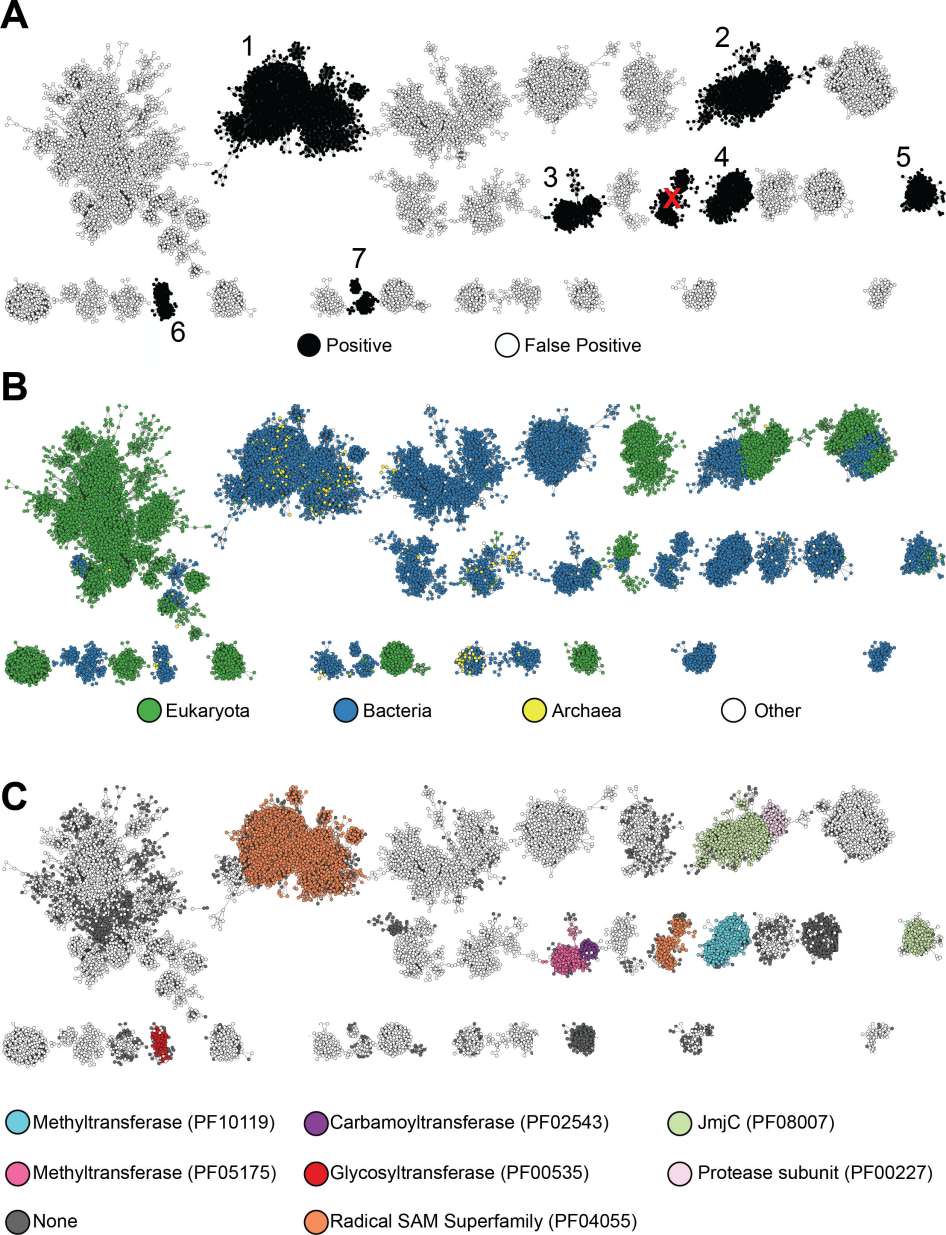
SSN of sequences retrieved by Foldseek but not RRE-Finder. The network represents 84,071 full-length UniProt sequences visualized at an alignment score of 30; groups with fewer than 100 nodes were removed for clarity. Node groups are numbered according to the Foldseek-derived HMMs (e.g., Foldseek_RRE_1 is derived from Group 1; see Table S1). The group marked with a red “X,” despite containing predicted RREs, was excluded due to poor HMM performance. (**A**) Groups containing predicted RREs (black) and false positives (white). (**B**) Taxonomic domain distribution based on UniProt annotation; “other” includes viral proteins or those lacking domain annotation. (**C**) Pfam distribution of annotated groups. Groups without RREs were only annotated if they shared a Pfam with a group containing a validated RRE. White nodes represent any Pfam not listed in the legend.

Upon viewing the data set of 99,650 putative RRE domains at RepNode 40, the number of unique nodes on the SSN was only reduced to 96,123, highlighting the diversity of retrieved sequences. Manual inspection of 100 randomly selected proteins from groups with 100 or more members revealed a very high false-positive rate of ~75% (Fig. 1). False positives were defined as proteins lacking the proper RRE βββ-ααα domain structure. Unfortunately, shorter protein domains deviating from this strict definition could still acquire high structural bitscores from FoldSeek. Their occurrence was independent of taxonomic origin. These findings demonstrate that while Foldseek enables broad identification of structurally similar proteins and discovery of novel RRE fusions, it struggles with discriminating amongst shorter domains, thus requiring extensive manual curation to separate high-confidence RREs from clear false positives.

We sought to enhance RRE-Finder by incorporating sequence-diverse proteins that were not retrieved by re-running either exploratory or precision mode. As such, we added 11 new Foldseek-derived HMMs to precision mode (Data set 1, Table S1). These models were built from SSN groups containing 100 or more members and high-confidence AlphaFold 3-predicted RRE folds (Fig. S3, predicted Template Modeling, pTM, score ≥0.60). Each group was further expanded iteratively with high-confidence, low bitscore putative RREs. This iterative approach, optimized for both specificity and non-redundancy, produced 11 non-overlapping HMMs meeting expected retrieval breadth and accuracy (Fig. S4). Collectively, these additions substantially improve coverage of previously unreachable RRE sequence space in precision mode, accounting for ~10% of entries in the updated precision mode (Data set 2).

### Reassessment and expansion of an updated data set of RRE domains

**Fig 2:**
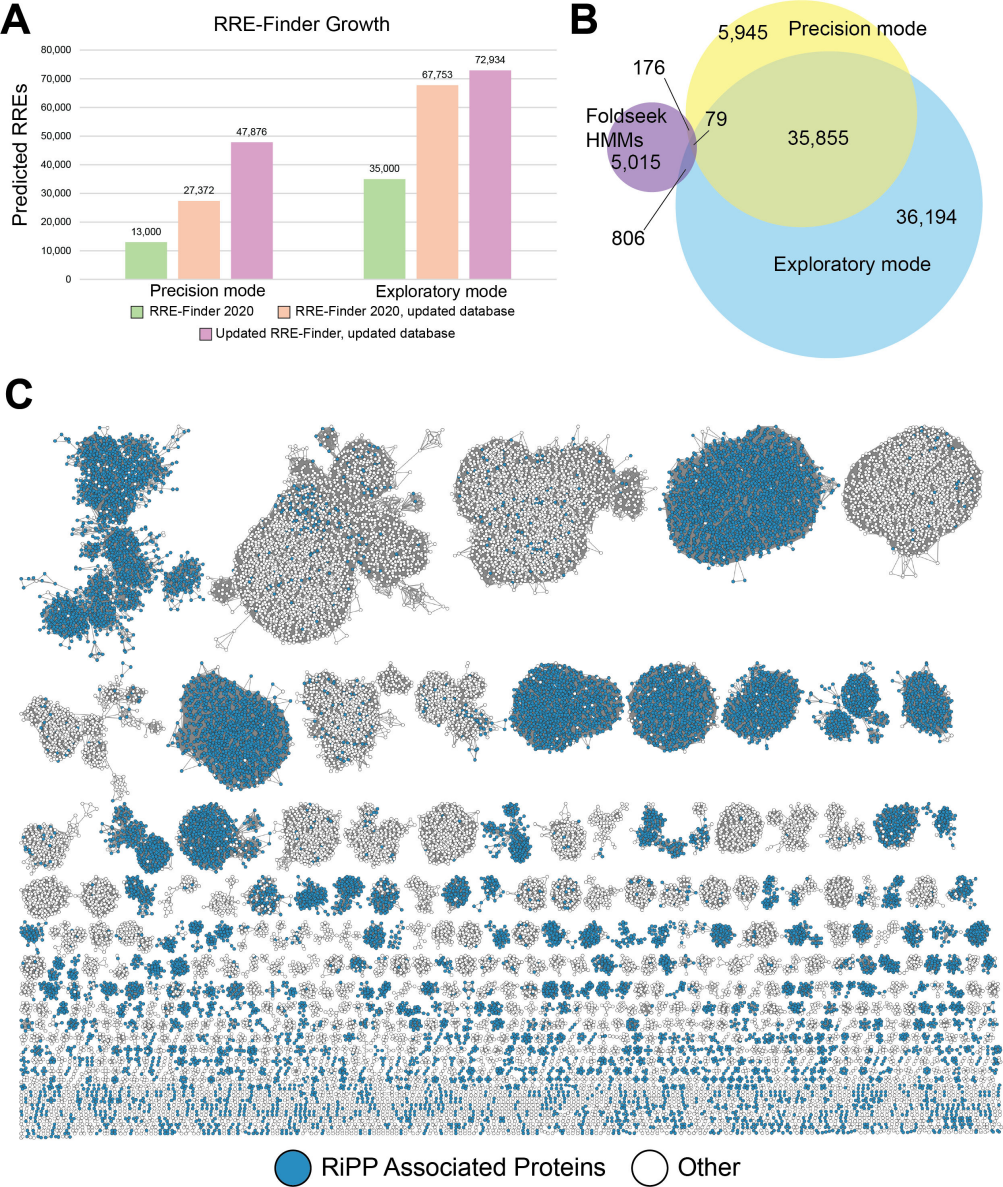
Overview of the complete RRE-Finder data set. (**A**) Comparison of data set growth relative to the original 2020 RRE-Finder release. Values are reflective of unique protein sequences. The updated precision mode includes all HMMs listed in Table S1. Re-run 2020 HMMs (excluding antiquated PlpY and auxiliary full-protein models) retrieve fewer sequences than the updated HMMs. (**B**) Venn diagram showing the overlap of unique sequences retrieved for the updated RRE-Finder. Foldseek-derived HMMs are shown as a separate set from the rest of the precision mode HMMs. (**C**) SSN of the RRE-Finder data set. Full-length UniProt sequences (*n* = 91,283) collapsed into 52,278 nodes (RepNode = 60), connected by 3.7M edges and visualized at alignment score = 60.

In addition to the Foldseek-derived HMMs, previously generated HMMs were evaluated for accuracy, model redundancy, and false-positive contribution from fused domain homology. We also provide model-specific bitscore cutoffs optimized for specificity and sensitivity. When necessary, overlapping models were applied to ensure coverage of sequences not adequately captured by a single HMM (Fig. S4). Since the original RRE-Finder was released in 2020, we have included new class-based HMMs from RRE-containing fusions discovered since the initial release of the algorithm. Accordingly, precision mode has been expanded from 29 to 59 total HMMs (Table S1). To reassess the scope of RREs retrieved, we re-ran precision and exploratory mode against the UniProtKB database and compared the number of proteins retrieved to the initial publication to evaluate both database growth and expansion of RRE-Finder. In 2020, precision mode retrieved ~13,000 unique sequences. Using the original 2020 HMMs, excluding antiquated models, and previously recommended bitscore cutoff = 25, we queried the updated UniProtKB and retrieved nearly twice as many proteins, highlighting database growth. Taking the updated HMMs and filtering metrics for precision mode and running them against the same database, we saw an additional 174% increase in the number of proteins retrieved, which was attributed to HMM enhancements. Repeating this with the exploratory mode, we also saw a small growth in proteins retrieved (Fig. 2). Combining the re-collected precision and exploratory mode data sets and applying previously described filtering metrics, we recovered 91,283 candidate RREs. When reduced to 100% identity, this corresponds to 84,070 unique protein sequences. Approximately 14% of these unique sequences were exclusive to precision mode, and 43% were unique to exploratory mode.

We next examined the phylogeny of all detected RRE domains. At the time of data set collection, 64% of all UniProt entries were from bacteria, whereas 93.5% of RRE domains were from bacteria (Fig. S5) (9, 21). However, ~1.5% of RRE domains were annotated as eukaryotic. We sought to determine if these were true RREs or false positives. Although there are characterized fungal RiPPs, none currently have known RREs (18, 37, 38). Closer inspection of putative RRE domains from fungi identified bacterial matches with >95% sequence identity, suggesting that the sequencing sample may have been contaminated with bacteria and misassigned as fungal (39). Inspection of the predicted structures from most metazoa and viridiplantae examples deviated from the required βββ-ααα order of secondary structure. Interestingly, a subset of fish and algae-derived RRE domains fused to an annotated JmjC domain (PF08007) displayed legitimate RRE-like folds (i.e., βββ-ααα) with BLAST results supporting eukaryotic origin. Given that genes are less frequently clustered by related functions in eukaryotes, it was unsurprising that no nearby precursor peptides were found; however, the closest JmjC bacterial homologs (~30% identity) appeared in RiPP-like genomic contexts (40–45). These observations suggest a strong possibility that RRE-like domains are present in all three domains of life.

Having analyzed their phylogenetic distribution, we next investigated the genomic neighborhood of the retrieved RRE domains (seven predicted genes upstream or downstream) (2). Approximately 60% of the RREs were either fused or proximally encoded to a well-characterized RiPP-related protein (Fig. 2). Common examples include, but are not limited to, cyclodehydratases, proteases, and rSAMs (Fig. S6) (5, 19, 20, 41, 46).

Beyond neighborhood-level associations, we examined the domain architectures of RRE-containing proteins. Fusion of an RRE to another protein domain suggests a functional partnership. RREs serve to recognize the leader region and deliver the substrate to the enzyme(s) but can also potentiate the activity of those enzymes (19). Upon the retrieval of 1,158 unique Pfam domains by RRE-Finder, we observed RREs fused to enzymes with precedence in RiPP biosynthesis, but not previously known to contain RREs, such as carbamoyltransferases (PF02543) and glycosyltransferases (PF00535) (47–50). The Pfam annotations suggest functions analogous to the assigned protein family; however, inference of function from homology can be misleading owing to evolutionary divergence. For example, the macrolactam-forming lasso cyclase common to all lasso peptide biosynthetic pathways is most often annotated as an asparagine synthetase (51–53). The remainder of retrieved proteins included high-confidence AlphaFold-predicted domain fusions unprecedented in RiPPs, such as Memo-like proteins (PF01875) and iron-sulfur cluster assembly proteins (formerly DUF59, PF01883) (54, 55). In addition to newly identified domain fusions, 21% of retrieved proteins lacked any Pfam match (Fig. S6). This highlights how RRE-Finder, particularly with the inclusion of new HMMs, can assist in placing uncharacterized or unannotated proteins into a biological context, reinforcing its utility for exploring RiPP diversity. Given that PF05402 was the most common domain annotation in our data set, we examined these proteins in greater detail. Among proteins annotated solely with PF05402, 71% were 110 or fewer amino acids in length, consistent with discrete RRE domains. This contrasts with the broader RRE-Finder data set, where ~80% of putative RREs exceeded 110 amino acids and are typically fused to an additional annotated domain. Discrete RREs were most frequently observed in lasso peptide and PQQ BGCs, although examples also appear in other RiPP classes, such as some sactipeptides (e.g., StsC, UniProt: A0A1R1S990) (56). Of the PF05402-annotated proteins, 6.9% had at least one additional Pfam annotation. Notably, 21% of the proteins matching PF05402 were above the discrete RRE size cutoff but had no other Pfam annotation (Fig. S6). This highlights a promising opportunity to explore uncharacterized proteins within RiPP-like BGCs, where the locally encoded precursor peptide provides a defined substrate for investigating novel enzyme chemistry (57).

### Prediction and mapping of recognition sequences

The recognition sequence is a crucial determinant as to whether a given peptide will be a substrate for the RiPP-modifying enzyme. Knowing the residues comprising the recognition sequence holds practical value for applications spanning the design of hybrid RiPPs to discovering novel RiPP precursor peptides (58–60). Given that experimental validation of recognition sequences requires labor-intensive mutational analysis, truncation studies, or protein structure determination (14, 46, 58, 61–67), we sought to develop a framework that allows accurate prediction of RiPP recognition sequences. Accordingly, we analyzed over 8,000 excised RRE–precursor peptide pairings from the expanded precision mode data set. Among these, we identified candidate RiPP BGCs and selected nearby genes as encoding putative precursor peptides using RODEO (8). Candidate precursor peptides were cataloged based on SSN group, presence of Pfam domains previously associated with RiPP biosynthesis, and conservation across homologous BGCs (2). If inspected RREs had ambiguous or inconsistent features, they were excluded to minimize false positives.

**Fig 3:**
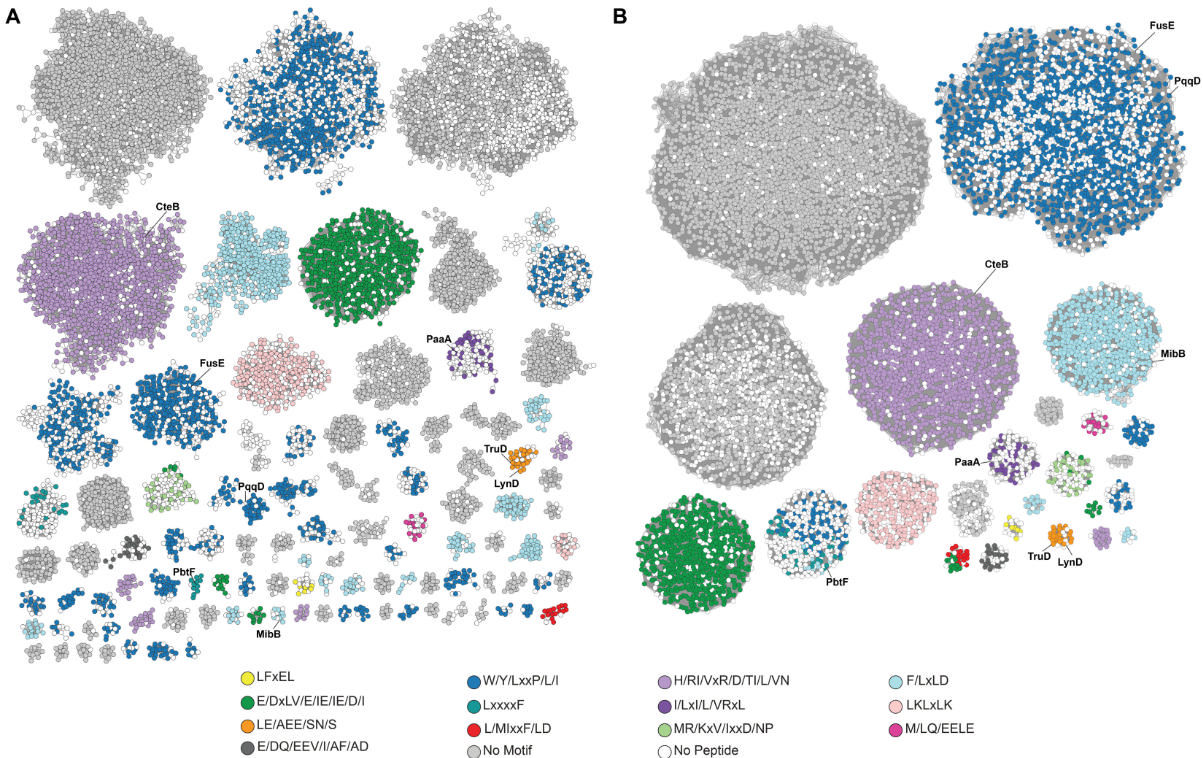
Recognition sequence mapping data set visualized as SSNs. (**A**) Recognition mapping SSN includes 44,350 RRE sequences represented as 11,118 nodes (RepNode = 90), visualized at an alignment score of 20. Coloring reflects conserved recognition sequences (*n* = 8,285 peptides identified across excised RREs). White nodes lack an identified precursor peptide. Several structurally characterized RREs are annotated for reference. (**B**) The same sequences are viewed at an alignment score of 10.

Alignments and sequence logos were generated for each RRE group, revealing 13 distinct and mostly RiPP class-specific motifs (Fig. 3). AlphaFold 3 was used to model RRE–peptide complexes (Fig. S6) (68). Conserved residues predicted to occupy the RRE binding cleft (formed by α3 and β3) were annotated as candidate recognition sequences. Several motifs have been previously characterized, including YxxP (lasso peptide), Lx_4_F (thiopeptide), HIxxI (sactipeptide), and FxLD (lanthipeptide), whereas the remainder await experimental validation (14, 64, 69, 70). Within these dominant motifs, most variation involved hydrophobic substitutions, such as YxxP to LxxP or FxLD to LxLD, indicating residue-level tolerance within conserved frameworks (61, 69).

Analysis of the phylogenetic and recognition sequence trends within the data set revealed new insights into the evolution of the RRE domain. For most groups, there is a strong correlation between phylogenetic origin, RiPP class, and recognition sequence (Fig. 3; Fig. S7). At an alignment score of 20, for example, there is a group of thiopeptide-related RREs from Actinomycetota that bind an Lx_4_F recognition sequence. Thiopeptides from Bacillota form a separate group that engages a different recognition sequence. When viewing the SSN at a more lenient alignment score of 10, these two thiopeptide groups merge. This case example illustrates that related RiPPs of the same class (i.e., thiopeptides) use more similar RRE domains despite differences in phylogenetic origin and recognition sequence.

Although the above suggests a certain evolutionary trajectory for RRE domains, it is not universal across all groups. To highlight a secondary trajectory, viewing the SSN at an alignment score of 20 shows many groups of RREs binding to a W/Y/LxxP/L/I recognition sequence. Among them are separate groups for FusE, McjB, PqqD, and StsC, which are involved in the biosynthesis of a lasso peptide from Actinomycetota, a lasso peptide from Pseudomonodota, PQQ from Pseudomonodota, and a sactipeptide from Actinomycetota, respectively (Fig. 3; Fig. S7) (14, 33, 56, 56). Unlike the above case, this particular recognition sequence is broadly distributed and does not appear to be specific to phylogenetic origin or RiPP class. Upon viewing the SSN at an alignment score of 10, these groups merge into a large, multiphyletic, and multi-RiPP class group. This finding suggests potential horizontal gene transfer of the RRE domain, as they are overall more sequence similar and bind to the related recognition sequences despite their distinct phylogenetic origins or RiPP class. Notably, FusE (discrete lasso peptide RRE) has greater sequence relatedness to PqqD (a non-lasso peptide, 60% similarity, 25% identity) than to the excised RRE domain of McjB (fused lasso peptide architecture, 54% similarity, 23% identity).

Although most groups exhibited recognizable motifs, the DUF2063-associated RREs (e.g., Protein Data Bank code: 3DEE), frequently paired with DUF2282 precursor peptides, displayed Sec-dependent signal peptides followed by variable leader regions, rather than a specific motif predicted to be recognized by an RRE (Fig. 3) (71–75). Altogether, this framework for recognition sequence mapping provides a scalable strategy for rapid precursor peptide identification and functional annotation. In addition to the evolutionary insights, the 13 motifs identified should facilitate systematic, higher-throughput substrate prediction across diverse RiPP classes, whereas examples like DUF2063 emphasize the continued need for complementary structural and experimental approaches when recognition sequences are ambiguous or absent.

### Validation of RRE-recognition sequence interactions

Following the identification of the recognition sequence through bioinformatic analyses and structural modeling, we sought to experimentally validate the predicted RRE–recognition sequence interactions. We selected RREs representing varying degrees of prior characterization. The most well-characterized case was the thiopeptide precursor TbtA (UniProt: D6Y501) and its associated RRE-containing protein TbtF (UniProt: D6Y506). Previous mutagenesis studies identified an Lx_4_F motif in the TbtA leader region (TbtA_Leader_) as key residues that mediate nanomolar affinity to TbtF_RRE_ (K_D_ ~70 nM) (70). These residues, specifically Leu(−29) and Phe(−24) which are negatively numbered using the convention established for RiPP leader regions, directly interact with key positions along α3 of the RRE (9, 76).

We attempted to crystallize the TbtA-TbtF complex but were unsuccessful and instead utilized the orthologous pair from *Planobispora rosea*, PbtA-PbtF (77). Sequence mapping of the recognition regions grouped PbtF’s RRE (UniProt: U5Q0A7) with that of TbtF, supported by overall high sequence similarity. TbtA and PbtA also share high sequence similarity (81% across 32 residues of the leader). A purified 13-residue leader peptide fragment composed of residues Asn(−33) to Ala(−21) of PbtA (UniProt: U5Q0A5, PbtA_13mer_), containing the Lx_4_F motif, was co-crystallized with the RRE of PbtF (PbtF_RRE_). The structure (PDB: 8T19) was solved at 1.23 Å resolution. The electron density enabled confident modeling of eight residues of the PbtA leader peptide, Leu(−29) to Met(−22), encompassing the recognition sequence (Fig. S8).

**Fig 4:**
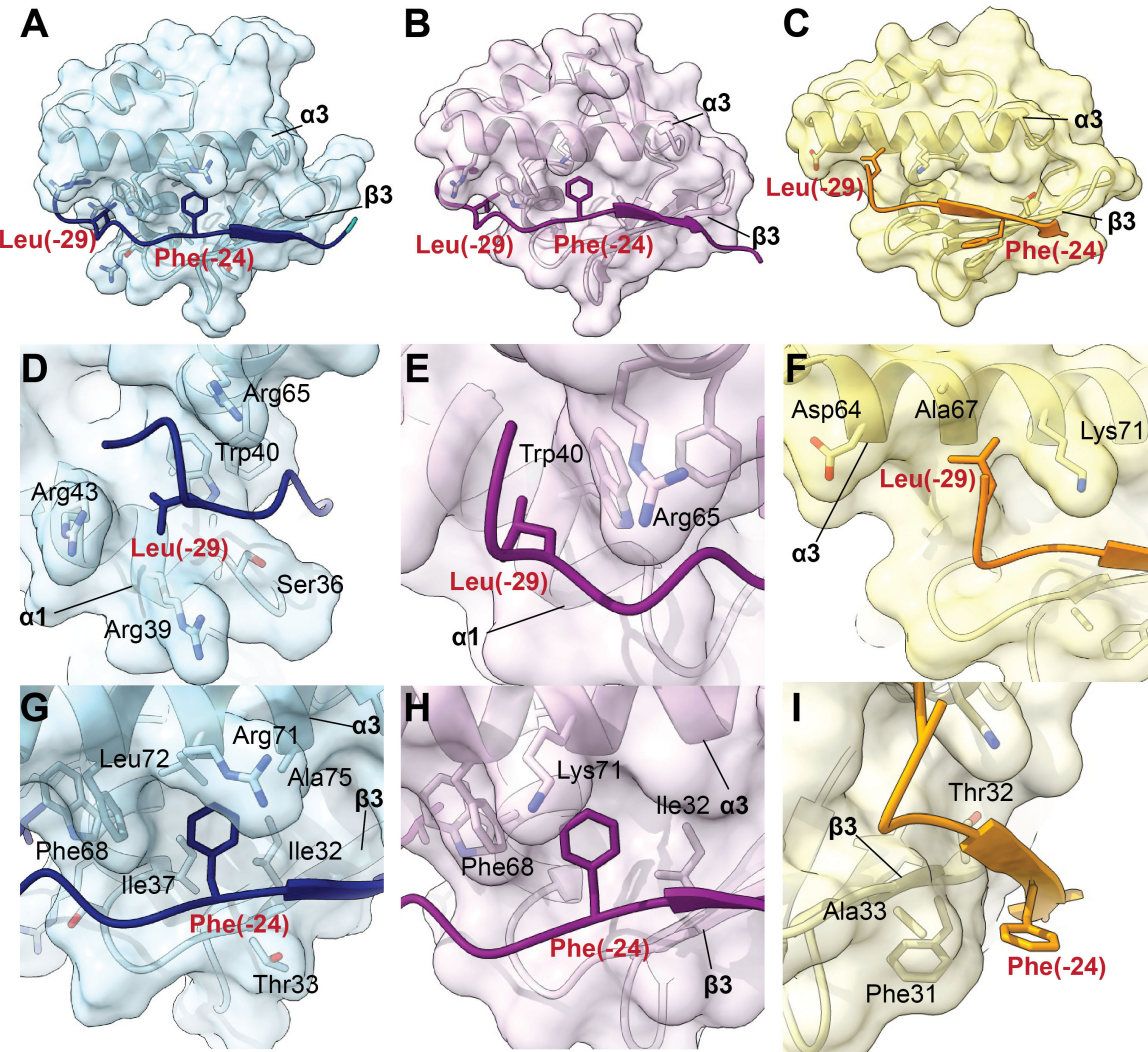
Comparison of AlphaFold and crystal structures of TbtF and PbtF bound to their respective leader peptides. RRE residues within 4 Å of the recognition residues are denoted in black text. (**A**) AlphaFold structure of TbtF_RRE_ with TbtA_Leader_ (blue) (ipTM: 0.74, pTM: 0.77). (**B**) AlphaFold structure of PbtF_RRE_ with PbtA_Leader_ (purple) (ipTM: 0.74, pTM: 0.79). (**C**) Crystal structure of PbtF_RRE_ with PbtA_13mer_ (yellow). (**D**) Zoom of panel A, Leu(−29) from TbtA_Leader_ occupies a predicted hydrophobic pocket near α1. (**E**) Zoom of panel B, Leu(−29) from AlphaFold 3 PbtA_Leader_ occupies a predicted hydrophobic pocket near α1. (**F**) Zoom of panel C, Leu(−29) from PbtA points toward α3 but does not occupy the predicted pocket. (**G**) Zoom of panel A, Phe(−24) from TbtA_Leader_ fits within a predicted cleft near α3. (**H**) Zoom of panel B, Phe(−24) from PbtA_Leader_ fits within a predicted cleft near α3. (**I**) Zoom of panel C, Phe(−24) from PbtA_13mer_ instead aligns with β3 and is positioned outside the cleft.

Comparing the PbtF_RRE_-PbtA_13mer_ crystal structure to the AlphaFold-predicted TbtF_RRE_-TbtA_Leader_ model showed close alignment (RMSD = 1.03 Å), indicating high structural similarity (68, 78). However, the predicted and experimental models diverged in peptide binding mode. In the AlphaFold 3 TbtF_RRE_-TbtA_Leader_ model, Leu(–29) and Phe(–24) of the peptide occupy defined pockets near α1 and along α3, respectively, consistent with mutational data identifying both residues as critical for RRE recognition and binding (Fig. 4). Notably, Phe(–24) engages in a π-stacking interaction with TbtF Phe68, with the F68A variant abolishing binding to TbtA_Leader_ (9).

AlphaFold 3 modeling of the PbtF_RRE_-PbtA_13mer_ complex predicted the same binding configuration as the TbtF_RRE_ model, consistent with functional data but differing from the crystal structure. In the PbtF_RRE_-PbtA_13mer_ crystal structure, Leu(–29) is solvent-exposed and Phe(–24) is oriented toward β3, potentially engaged in a π-stacking interaction. This arrangement contrasts with the mutational data and the AlphaFold 3 model, which place these residues in defined hydrophobic pockets along α1 and α3, consistent with key recognition roles. Notably, because the structure of PbtF_RRE_ was solved in isolation from the full-length protein, the observed binding mode may be influenced by structural truncation or by crystallographic contacts between asymmetric units, potentially perturbing its native binding conformation. Taken together, these results suggest that the AlphaFold 3 model likely provides a more accurate representation of the native RRE–peptide binding mode for PbtF than the crystal structure.

To further confirm or refute the accuracy of the AlphaFold-predicted interactions, we selected a second test case. We streamlined the experimental workflow by utilizing a double-Ala scanning strategy, in which adjacent residues were substituted in pairs to Ala. To accommodate the higher number of variants, binding was detected using affinity co-purification with immobilized RRE proteins, followed by LC-MS detection, as it provided an automated, rapid, and sensitive readout (Fig. S9). We applied this approach to investigate the StsA_Leader_–StsC (RRE) interaction, as StsC was predicted to bind the WxxP recognition sequence of StsA (NCBI: WP_158080382.1) (56). As positive controls, wild-type StsA and an E(−11)A/M(−10)A double variant, located outside the predicted recognition sequence, were retained during affinity co-purification with immobilized StsC. The variants containing W(−15)A/V(−14)A and K(−13)A/P(−12)A were not retained (Fig. S9). Unexpectedly, the K(−18)A/P(−17)A variant also abolished binding, despite these positions not being well conserved (56). AlphaFold 3 predicts the side chain of Lys(−18) to point towards Glu62 and Asp63 situated in the loop between α2 and α3 of the RRE, suggesting a potential interaction. Pro(−17) is also within 4 Å of the side chains of Arg58 and Tyr59 on α2, expanding the proposed recognition sequence to KPxWxxP. With only one known exception (i.e., SuiB, Protein DataBank 5VIT), the recognition sequence of the leader peptide adopts a β-strand conformation and engages β3 of the RRE, which is reflected with high confidence in the predicted complex (14, 46, 62).

**Fig 5:**
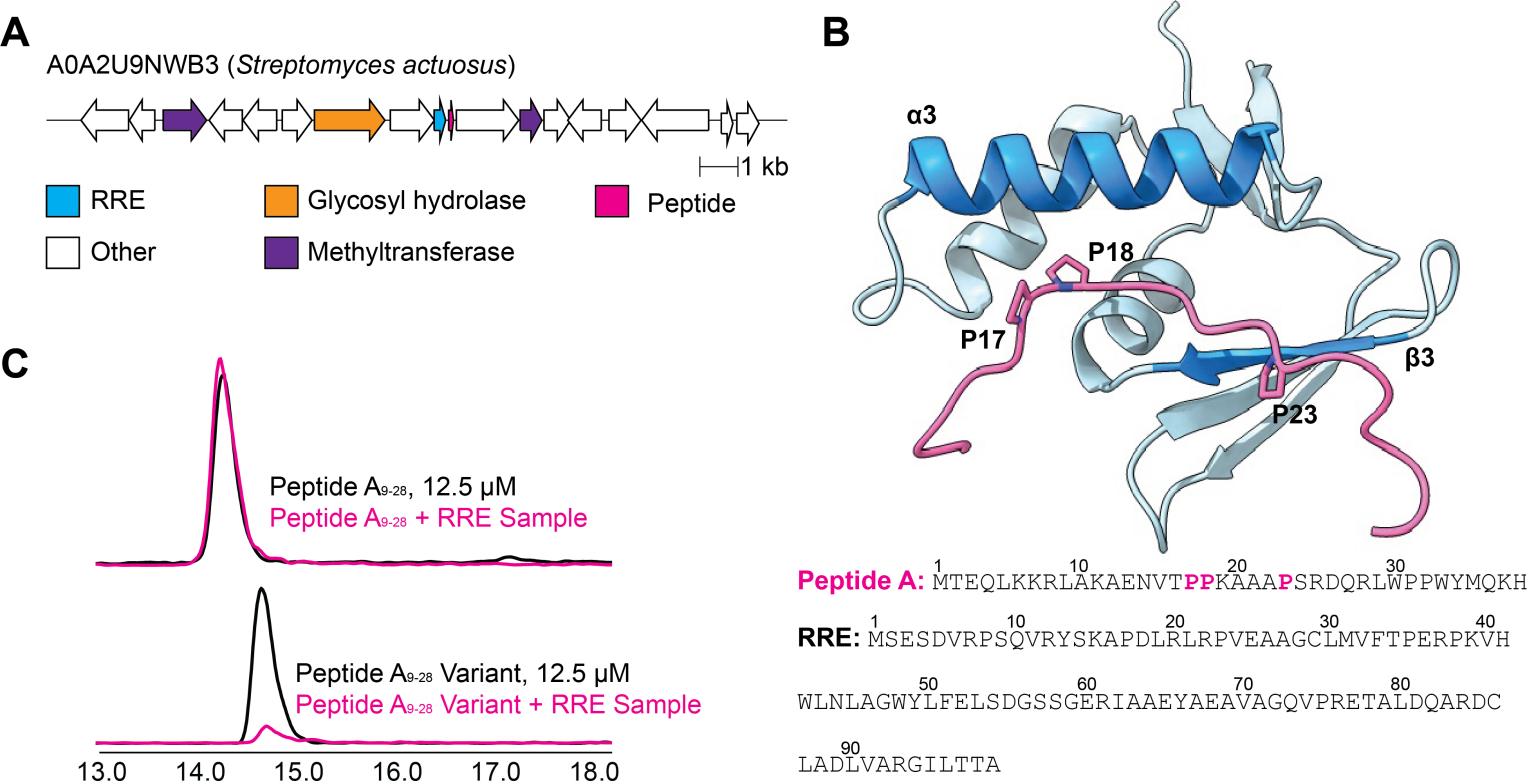
Identification and validation of a new RRE–peptide interaction via AlphaFold 3 prediction. (**A**) BGC diagram showing local genomic context for an RRE-containing protein (UniProt: A0A2U9NWB3); the very short, unannotated putative precursor peptide is indicated in pink (74). (**B**) AlphaFold 3-predicted complex between RRE (blue) and its putative peptide (all residues, trimmed for visualization, pink, ipTM: 0.68, pTM: 0.77), with the predicted recognition sequence in bold. (**C**) LC–MS peptide capture assay comparing Peptide A_9-28_ (residues 9–28 of Peptide A) and a triple Ala variant (P17A/P18A/P23A). Pink traces show experimental data for Peptide A_9-28_ and the triple-Ala variant peptide, scaled to the same axis; black traces correspond to peptide standards used for retention time confirmation. EIC traces are shown for [M + 4H]^4+^ ± 0.2 Da. Peptide A_9-28_ (top): 597.65 Da. EIC traces are shown for [M + 3H]^3+^ ± 0.2 Da. Peptide A_9-28_ variant (bottom): 757.25 Da.

As a more stringent test, we examined whether AlphaFold could predict RRE–recognition sequence interactions that were not mapped. We filtered the RRE-Finder data set for discrete RREs within RiPP BGCs, then modeled the RRE against all possible encoded peptides that lacked known recognition sequences. From these candidates, we selected an uncharacterized RRE from *Streptomyces actuosus* (UniProt: A0A2U9NWB3), which is encoded near a predicted methyltransferase, glycosyl hydrolase, and an unannotated putative precursor peptide (Fig. 5) (2, 5, 79–81). AlphaFold 3 modeled a Pro-rich region of the peptide (Peptide A) into the RRE binding cleft, representing a previously unknown recognition sequence. Using the peptide capture assay, wild-type Peptide A was retained by the RRE, while the triple Pro variant (P17A/P18A/P23A) significantly reduced binding (Fig. 5). Compared with the traditional approach, this procedure of identifying recognition sequences is considerably less time-consuming.

Given our results on PbtF-PbtA, our work raises the question of when an AlphaFold-predicted peptide-bound RRE structure may be more reliable than an experimentally determined structure. The ligand placement accuracy of AlphaFold has been broadly examined, with published examples showing where the algorithm performs equivalently to experimental data but others where its performance suffers (82–86). Furthermore, there are known cases where AlphaFold augments incomplete crystal structures owing to a lack of electron density in flexible regions. For instance, several deposited ThiF–ThiS structures (and the analogous MoeB–MoaD) omit the catalytic Cys184 due to missing density, whereas AlphaFold (UniProt: P30138) correctly positions this residue near the ATP-binding site (87, 88).

Together, these findings underscore how AlphaFold 3 can streamline both recognition-sequence prediction and structural analysis in RiPP pathways. To our knowledge, this study represents one of the few cases where an AlphaFold model more accurately reflects a binding interface than a solved structure. In this context, AlphaFold serves as a useful tool to guide biophysical characterization and accelerate RiPP discovery.

### Conclusions

In this study, we expanded the RRE-Finder framework by integrating structure-guided searches with Foldseek, building new sequence-diverse HMMs, and validating RRE–peptide interactions through AlphaFold 3 modeling and *in vitro* binding assays. These efforts substantially increased the coverage of RRE sequence space, reduced false positives, and enabled the mapping of 13 distinct recognition sequences. Together, these improvements demonstrate how structural information can sharpen genome-mining strategies and streamline precursor peptide identification across RiPP classes.

The updated precision mode is not only more accurate than the previous iteration but also recovers 174% more proteins when re-run on the current database compared with the original. The addition of Foldseek-derived HMMs to precision mode contributed entirely novel domain fusions. These findings point to underexplored enzymatic activities in RiPP contexts and suggest that leader/follower peptide–guided chemistry is more versatile than previously appreciated. Moreover, the identification of RRE-like folds in metazoan proteins raises the very real possibility that this recognition strategy extends to higher eukaryotes.

Systematic mapping of recognition sequences across 8,000 RRE–precursor peptide pairs supported by AlphaFold 3 enabled the identification of 13 distinct motifs and revealed subtle residue-level flexibility within otherwise conserved frameworks, providing a more detailed view of RRE–recognition sequence interactions. In addition, the sequence similarity analysis overlaid with phylogenetic origin and recognition mapping revealed that RRE domains have taken multiple evolutionary trajectories. Comparisons to *in vitro* data demonstrated that AlphaFold predictions reliably captured biologically relevant RRE–RS interactions, highlighting its utility as a complementary tool for putative substrate identification and minimal substrate design.

Looking forward, the combined tools and data sets described here help expand the foundation for both discovery and engineering. Structural models of RRE–precursor peptide interactions can accelerate substrate assignment in uncharacterized BGCs, whereas the broadened catalog of RRE fusions offers new entry points to interrogate enzymatic novelty. More broadly, these strategies illustrate how structure-guided genome mining can reveal hidden diversity in small-molecule biosynthesis and set the stage for future efforts to engineer RiPP pathways toward new scaffolds and optimized bioactive compounds.

## MATERIALS AND METHODS

### General materials and methods

Reagents used for molecular biology experiments were purchased from New England Biolabs (NEB, Ipswich, MA), Thermo Fisher Scientific (Waltham, MA), or Gold Biotechnology Inc. (St. Louis, MO). *Escherichia coli* DH5α and BL21(DE3) strains were used for plasmid maintenance and protein overexpression, respectively. Oligonucleotides were purchased from Integrated DNA Technologies Inc. (Coralville, IA). Genes optimized for recombinant expression in *E. coli* were synthesized by Twist Bioscience (South San Francisco, CA)**.**

### Structural prediction of RREs

Structural predictions of RRE domains with and without substrates were predicted using the AlphaFold 3 web server (32).

### Foldseek data set generation

To evaluate bitscore thresholds for Foldseek, we used PqqD (PDB: 5SXY) as an initial query under the exhaustive search setting. AlphaFold 3-predicted structures were manually inspected, and a bitscore cutoff of 40 was selected based on an estimated 87% accuracy. For broader mining, random RRE representatives from previous RRE-Finder data were excised and modeled with AlphaFold 3 to assess if the canonical RRE fold was confidently predicted. Candidate proteins were filtered as follows: (i) by bitscore (≥40 were retained, resulting in 137,726 proteins), (ii) for length (proteins ≥65 amino acids were retained, resulting in 118,769 proteins), and (iii) by removing entries that represent known false-positives. This latter step included manual inspection of RRE domains identified by exploratory mode (196 Pfam identifiers have been flagged as false positives, see Supplementary File Pfam filtering list, 99,650 proteins retained) (31).

### Precision mode HMM generation

New Foldseek-derived HMMs were created from the largest Foldseek groups (≥100 nodes) containing AlphaFold 3-predicted RRE structures with pTM scores ≥ 0.60. For each group, the RRE domain was excised using HHpred or AlphaFold 3 and used to seed a BLAST search (89). A subset of 5–20 sequences per group was selected for multiple sequence alignment and used to generate the HMM using HMMER (22, 32). To increase model diversity, low-bit-score proteins for Foldseek-derived HMMs not covered by existing HMMs were included as seeds for additional HMMs. All HMMs are provided in Data set 1 and integrated into the RODEO web tool (https://webtool.ripp.rodeo/) (8).

### Validation of precision mode HMMs

HMM results were evaluated for precision and accuracy by running all accession IDs through HHblits (90) to search for homologs using the following structures: 6JX3, 5V1V, 5V1T, 5SXY, 3DEE, 4V1T, 4BS9, 5LQ4, 5EHK, 4WD9, 6M7Y, 6GOS, 6OM4, 5FF5, 5WGG, 6EFN, 6EC7, 8VPO, and 8HKR. Due to the large number of proteins, any degree of homology to the structures was considered a positive hit. Remaining proteins were manually inspected for typical RiPP BGC architecture (e.g., structurally predicted RRE domain, transporter, modifying enzymes). HMM-specific recommended bitscore thresholds were determined by evaluating precision, recall, and F1 plots (defined below).


(1) $$Precision=\;\frac{True\;Positive}{True\;Positive\;+\;True\;Negative}$$

(2) $$Recall\;=\frac{True\;Positive}{True\;Positive\;+\;False\;Negative}$$

(3) $$f1=2*\left(\frac{Precision\;*\;Recall}{Precision\;+\;Recall}\right)$$

### Exploratory mode generation

The exploratory mode was installed from the RRE-Finder GitHub (https://github.com/Alexamk/RREFinder) and run with the default settings against the entirety of UniProt KB with a bitscore cutoff of 25 and a probability cutoff of ≥90%.

### Pfam filtering list generation

To identify Pfam families to exclude as false positives, we retrieved all Pfams detected in exploratory mode. Pfams with at least 100 members were manually inspected by analyzing AlphaFold 3-predicted structures. If no RRE-like fold was identified after examining 100 representative proteins, the Pfam family was flagged for removal.

### RiPP-associated protein filtering

Genomic neighborhoods—defined as seven predicted genes upstream or downstream of the query protein—were searched for RiPP biosynthetic identifiers: PF00733 (lasso cyclase), PF02222 (ATP-grasp), PF04297 (PatG), PF04738 (LanB), PF14028 (LanC), PF00881 (SagB), IPR023850 (MftB), IPR011842 (PqqB), rSAM (PF04055), YcaO (PF02624), or MNIO (PF05114) domains. White nodes represent putative RREs lacking any of the above biosynthetic enzymes within the defined neighborhood.

### Recognition sequence mapping data set generation

RREs were filtered for the presence of easily recognizable enzymes known to be involved in RiPP biosynthesis, as of the most recent comprehensive review, within 7 ORFs (2). Corresponding hit RREs were then excised based on HMM alignment using a previously developed script (91). These excised RREs were submitted to EFI-EST to generate an SSN using an alignment score = 20 and a RepNode threshold = 90. Groups containing fewer than 10 nodes were excluded from further analysis. Remaining RREs were processed through RODEO to retrieve candidate precursor peptides, with an emphasis on genes bearing Pfam annotations commonly associated with RiPP precursors (e.g., PF23709 for mycofactocin or PF24178 for lasso peptides) (8). If annotated peptides constituted at least 50% of a group, the group was retained. For each RRE group, if at least 50% of precursor peptide candidates were retrieved, the group was retained. For groups below this threshold after using RODEO, additional precursor peptides were identified through manual inspection of conserved genomic neighborhoods, focusing on gene grouping behavior, sequence conservation across homologous BGCs, and contextual biosynthetic features. Groups lacking sufficient confidence assigned precursor peptides (*n* < 50% of group) or exhibiting inconsistent precursor peptide features were excluded from downstream recognition sequence analysis. Randomly selected representative RRE-peptide per group was predicted by AlphaFold 3 to see if the bioinformatically determined recognition sequence was also predicted to interact with the binding cleft of the RRE.

### MBP-tagged RRE overproduction and purification

*E. coli* BL21 (DE3) cells were transformed with a pET-28 plasmid encoding the maltose-binding protein (MBP)-tagged wild-type RRE. Cells were grown for 18 h on lysogeny broth (Miller) (LB) agar plates containing 50 µg/mL kanamycin at 37°C. Single colonies were picked to inoculate 10 mL of LB containing 50 µg/mL kanamycin and grown at 37°C for 16–18 h. This culture was used to inoculate 1 L of LB containing 50 µg/mL kanamycin and grown to an optical density OD_600_ of 0.8, then placed on ice for 30 min. Protein expression was induced by adding 100 µM isopropyl β-D-1-thiogalactopyranoside (IPTG). Expression proceeded for 3 h at 37°C. Cells were harvested by centrifugation at 4,500 × *g* for 20 min, washed with phosphate-buffered saline (PBS; 137 mM NaCl, 2.7 mM KCl, 10 mM Na_2_HPO_4_, 1.8 mM KH_2_PO_4_), and subjected to centrifugation at 4,000 × *g* for 20 min.

Harvested cells were resuspended in lysis buffer (50 mM Tris-HCl, pH 7.5, 0.5 M NaCl, 2.5% glycerol [vol/vol], 0.1% [vol/vol] Triton X-100) containing 4 mg/mL lysozyme, 2 µM leupeptin, 2 µM benzamidine HCl, 2 µM E64, and 30 mM phenylmethylsulfonyl fluoride. Cells were lysed by sonication (3 × 40 s with 10 min equilibration periods at 4°C with rocking). Insoluble cell debris was removed by centrifugation at 20,000 × *g* for 60 min at 4°C. The clarified cell lysate was then applied to amylose resin (NEB; 5 mL of amylose resin per L of cell culture) pre-equilibrated with lysis buffer. The column was washed with 10 column volumes (CVs) of lysis buffer followed by 10 CVs of MBP wash buffer (50 mM Tris [pH 7.5], 0.5 M NaCl, 2.5% [vol/vol] glycerol). The MBP-tagged proteins were eluted using 5 CVs elution buffer (50 mM Tris, [pH 7.5], 0.3 M NaCl, 10 mM maltose, 2.5% [vol/vol] glycerol). Eluent was concentrated using a 30 kDa Molecular Weight Cutoff Amicon Ultra centrifugal filter (EMD Millipore). A buffer exchange with 10× volume of protein storage buffer (50 mM HEPES, pH 7.5, 300 mM NaCl, 2.5% glycerol [vol/vol], 0.5 mM TCEP) was performed before the final concentration and storage. Purity was inspected by sodium dodecyl sulfate polyacrylamide gel electrophoresis (SDS-PAGE) and staining by Coomassie Brilliant Blue. Protein concentrations were assayed by bicinchoninic acid assay, and proteins were stored at −80°C until use.

### Protein crystallization

PbtF_RRE_ was purified by a combination of nickel affinity and size exclusion chromatographic methods. A purified 13-residue leader peptide fragment of PbtA (PbtA_13mer_) was purchased from GenScript (sequence: NLNDLPMDVFEMA). Purified PbtF_RRE_ was concentrated to 8 mg/mL using a 3 kDa Molecular Weight Cutoff Amicon Ultra centrifugal filter (EMD Millipore) and then incubated with 1.5 equivalents of PbtA_13mer_ in 20 mM HEPES, pH 7.5, 0.3 M KCl for 30 min in an ice bath. A single microliter of the PbtF_RRE_ + PbtA_13mer_ mixture was incubated with an equal volume of crystallization solution containing 27.5% PEG 3350, 0.2 M MgCl_2_, and 0.1 M tris, pH 8.0, and suspended on a glass slide above 500 µL of the same crystallization solution using the hanging drop crystallization method. Crystallization drops were incubated at 9°C, and crystals appeared within 24 h.

### Crystallographic structure determination and model building

Crystals of the PbtF_RRE_ + PbtA_13mer_ complex were (i) transferred into 2 mL drops of crystallization solution (above) supplemented with 1 mM EtHgPO_4_, (ii) incubated at room temperature for 30 min, (iii) briefly immersed in crystallization solution supplemented with 10% ethylene glycol, (iv) mounted onto nylon loops, and (v) promptly vitrified in liquid nitrogen. Diffraction data were collected at the synchrotron beamline 21-ID-F of the Advanced Photon Source at Argonne National Laboratory (Fig. S8). The structure was determined using single-wavelength anomalous diffraction collected at the Hg absorption edge. *Phenix AutoSol* was used to determine crystallographic phases and produce a preliminary atomic model rebuilt in *Phenix AutoBuild* (92, 93). The model was refined manually in *Coot* and automatically in *Phenix Refine* (94, 95). Diffraction data from native crystals that diffracted to 1.23 Å (Fig. S8) were phased using molecular replacement in *Phenix Phaser-MR* from the above coordinates and refined as above to produce the final deposited map and coordinate files (96).

### Leader peptide capture assay

N-terminally biotinylated peptides (Genscript) were dissolved in 1 mL dimethyl sulfoxide (DMSO) to a final concentration of 0.5 mg/mL. A 300 µL slurry of 50% amylose resin (in 20% ethanol) was loaded into Micro Bio-Spin Columns (Bio-Rad) and washed under vacuum with five column volumes (CVs) of MBP wash buffer using a Visiprep 12 manifold (Supelco). Next, 1 mL of MBP wash buffer containing 7.5 µM MBP-RRE was added to the resin and equilibrated for 15 min, with gentle pipetting every 5 min to ensure proper mixing. The resin was then washed three times with 1 mL MBP wash buffer under vacuum to remove unbound protein. Following this, 1 mL of peptide solution (15 µM), containing the recognition sequence of interest, was applied to the resin and incubated for 15 minutes to allow binding. The column was washed with three CVs of MBP wash buffer, followed by one CV of 50 mM Tris buffer (pH 7.5). To cleave the bound proteins, 300 µL of tobacco etch virus (TEV) protease solution (4.9 µM in 50 mM Tris, pH 7.5) was added and incubated for 1 h with gentle pipetting every 30 minutes to resuspend the resin. The RRE, peptide, and TEV protease were eluted by centrifugation at 500 × *g* for 1 min. The eluent was then incubated with 200 µL of 50% slurry nickel-nitrilotriacetic acid (Ni-NTA) resin (in 20% ethanol) for 15 min, and the cleaved RRE-peptide complex was eluted again by centrifugation at 500 × *g* for 1 min. Finally, the collected eluent was dried and resuspended in 100 µL of buffer containing 5% acetonitrile with 0.1% formic acid and 95% 50 mM tris with 0.1% formic acid.

Peptide binding was assessed by liquid chromatography–mass spectrometry (single-quadrupole, LC-MS, Shimadzu LCMS 2020). The most abundant charge state and retention time for each StsA peptide was determined using 5–15 µL injections of 12.5 µM peptide stocks onto a Hypersil Gold C18 column (100  ×  2.1  mm, Thermo Fisher Scientific) using mobile phase A (water, 0.1% FA); mobile phase B (ACN, 0.1% FA); gradient - 5 min at 5% B, 10 min 5%–10% B, 7 min 10%–40% B, 3 min 40%–60% B, 2 min 60%–90% B; flow rate - 0.3 mL/min. Total ion chromatograms were monitored from 5 to 30 min in a positive mode. Peptide capture samples (15–30 µL injections) were confirmed based on retention time overlap and extracted ion chromatograms (EICs) of the most abundant charge state. The same process was repeated for Peptide A and its corresponding RRE sample and standards, but the LC method was adjusted to 5 min at 5% B, 12 min 5%–40% B, 3 min 40%–60% B, and 2 min 60%–90% B.

## SUPPLEMENTAL MATERIAL

Supplemental material msystems.01252-25-s0001.pdf Supplemental figures and table.

ASM does not own the copyrights to Supplemental Material that may be linked to, or accessed through, an article. The authors have granted ASM a non-exclusive, world-wide license to publish the Supplemental Material files. Please contact the corresponding author directly for reuse.
