# Supplementary material for: Structure-based discovery and definition of RiPP recognition elements
Source: mSystems. 2025 Nov 18;10(12):e01252-25. doi: 10.1128/msystems.01252-25 (PMC12710340; doi:10.1128/msystems.01252-25)
Supplement: Supplemental material — Supplemental figures and table. [file msystems.01252-25-s0001.pdf]

Supplementary Information for

Structure-Based Discovery and Definition of RiPP Recognition Elements

Authors: Miriam H. Bregman[a], Dillon P. Cogan[b], Kyle E. Shelton[a], Andrew J. Rice[c], Shravan R. Dommaraju[c], Satish K. Nair[b,d,e], Douglas A. Mitchell[c,f,#]

Author Affiliations

[a]Department of Chemistry, Roger Adams Laboratory, University of Illinois at Urbana-Champaign, Urbana, Illinois, USA

[b]Department of Biochemistry, University of Illinois at Urbana-Champaign, Urbana, Illinois, USA

[c]Department of Biochemistry, School of Medicine – Basic Sciences, Vanderbilt University Medical Research Building-IV, Nashville, Tennessee, USA

[d]Carl R. Woese Institute for Genomic Biology, University of Illinois at Urbana-Champaign, Urbana, Illinois, USA

[e]Center for Biophysics and Quantitative Biology, University of Illinois at Urbana-Champaign, Urbana, Illinois, USA

[f]Department of Chemistry, Vanderbilt University, Medical Research Building-IV, Nashville, Tennessee, USA

#Address correspondence to Douglas A. Mitchell, douglas.mitchell@vanderbilt.edu

Present address: Dillon P. Cogan, Department of Pharmacology and Pharmaceutical Sciences, University of Southern California, Los Angeles, California, USA

Table of Contents

Figure S1: Structural comparison of RRE domains and other peptide-binding related folds S3

Figure S2: Statistics from the PqqD-derived Foldseek test dataset and input composition of the final Foldseek dataset. S4

Figure S3: Representative RRE-containing proteins from Foldseek used for Foldseek-derived HMMs. .. S5

Table S1: Recommended performance thresholds for RRE-Finder Hidden Markov Models (HMMs). S6

Figure S4: Matrix showing the overlap of all proteins scoring at or above the recommended bitscore cutoffs after filtering. S8

Figure S5: Investigation of putative eukaryotic RRE-containing proteins. S9

Figure S6: Breakdown of RRE-Finder Pfam distribution and PF05402 analysis. S10

Figure S7: AlphaFold 3-predicted RRE–peptide complex structures representing distinct recognition sequence groups and phylogenic distribution. S11

Figure S8: Crystallographic information and interactions between the RRE of PbtF (PbtFRRE) and its leader peptide. S12

Figure S9: StsA-StsC leader peptide capture assay and results. S13

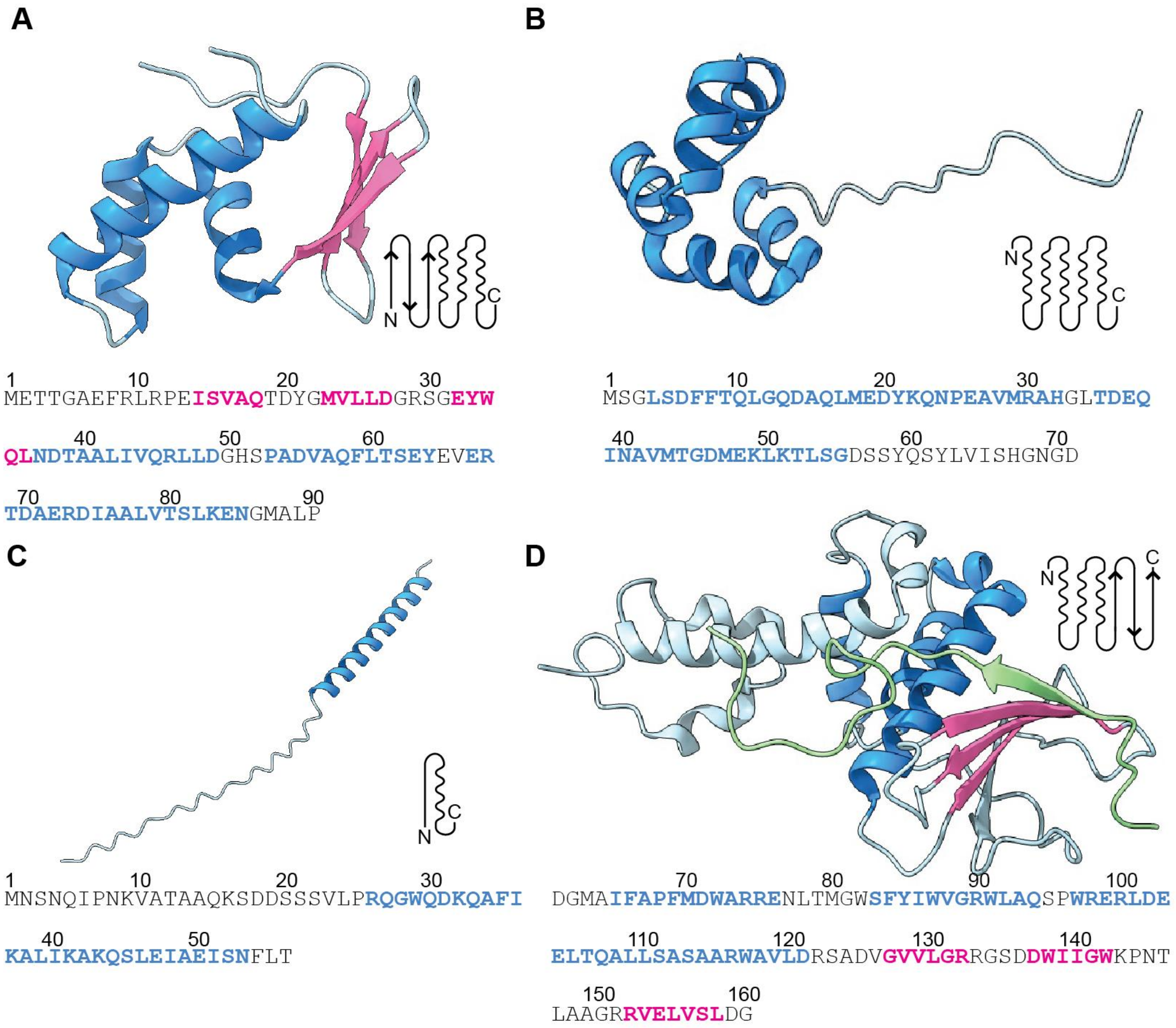

Figure S1: Structural comparison of RRE domains and other peptide-binding related folds. Alpha-helices are shown in dark blue and β-strands in pink. All structures were visualized using ChimeraX.[51] A) FusE (UniProt: Q47QT5, PDB: 6JX3). B) Crystal structure of SonA (UniProt: A0AAX2LRH2, PDB: 7LTE) showing the five helical bundle of the borosin binding domain. C) AlphaFold 3 predicted structure of PlpY (NCBI: WP_019503879.1, predicted Template Modeling score (pTM) = 0.38), too small to adopt the canonical RRE fold. D) Crystal structure of MbnC, which displays a reversed secondary structure arrangement, with the precursor peptide MbnA (green) bound (UniProt: A0A1I4IFH0, PDB: 7FC0).[19–22]

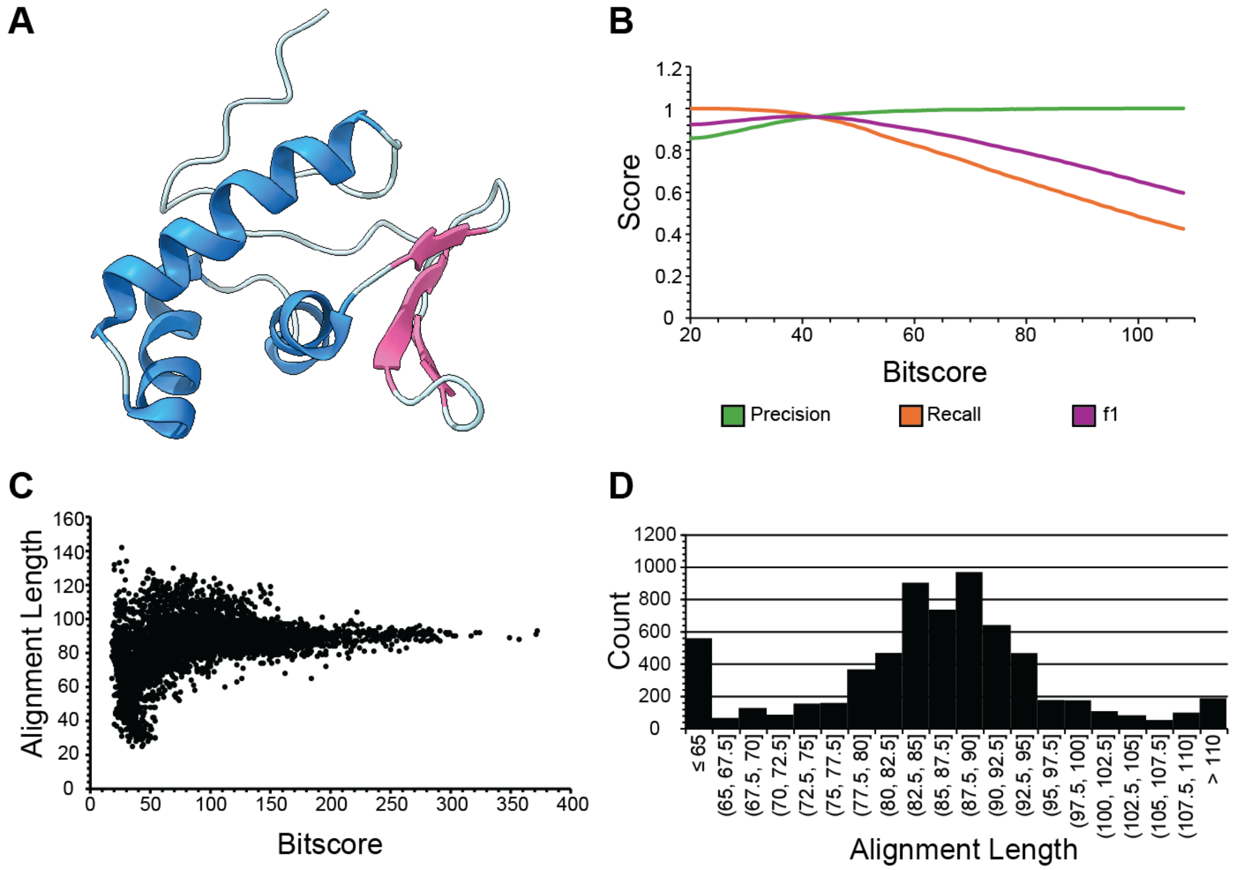

Figure S2: Statistics from the PqqD-derived Foldseek test dataset and input composition of the final Foldseek dataset. A) Input structure used for benchmarking: PqqD (PDB: 5SXY). B) Precision, recall, and f1 score plotted as a function of bitscore cutoff. C) Relationship between alignment length and bitscore across Foldseek-retrieved proteins (n = 6,670 proteins). D) Histogram of alignment lengths for proteins aligned to the PqqD structure (n = 6,670).

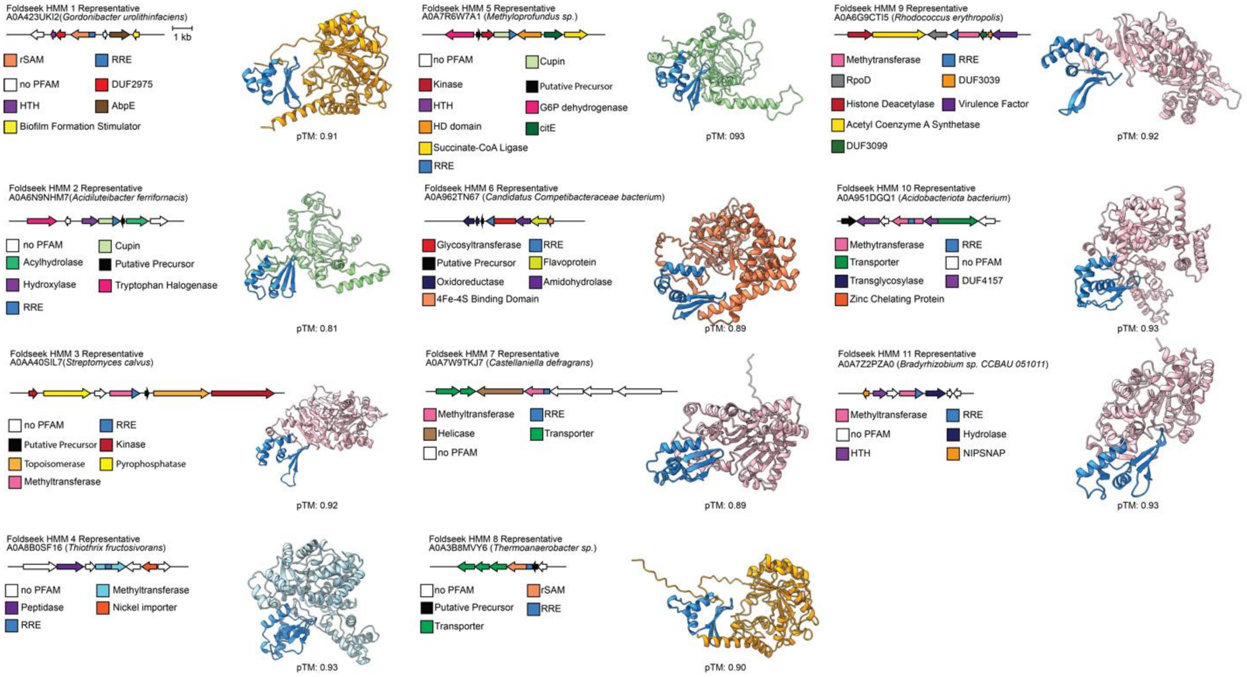

Figure S3: Representative RRE-containing proteins from Foldseek used for Foldseek-derived HMMs. BGC diagram showing 3 ORFs from RRE used as input for HMM. AlphaFold 3 prediction and pTM score for each input RRE with the RRE highlighted in blue.

Table S1: Recommended performance thresholds for RRE-Finder Hidden Markov Models (HMMs) for end users. The table reports the percentage of each dataset identified as true positives, along with the recommended bitscore cutoff for each HMM used in precision mode for users. HMMs shown in **bold blue** indicate newly added or improved models in this version of RRE-Finder. *HMMs without a designated class name are labeled according to the domain the RRE is fused to. **Represents HMMs that include multiple RiPP products that do not have a defined class name, such as bufferins and oxazolins.[19,55,64]

RREFam #	HMM Name	% True Positive	Recommended Bitscore Cutoff
RREFam001	**Aminopyruvatide_RRE_1**	92	19
RREFam002	**Aminopyruvatide_RRE_2**	25	30
RREFam003	Bottromycin_RRE	66	21
RREFam004	Cyanobactin_RRE	85	19
RREFam005	**Cyclopropyl_RRE***	48	21
RREFam006	**Daptide_RRE_1**	83	20
RREFam007	**Daptide_RRE_2**	72	20
RREFam008	**Daptide_RRE_3**	40	25
RREFam009	**Daptide_RRE_4**	55	20
RREFam010	**DUF5825_RRE**	86	25
RREFam011	Lanthipeptide_RRE_1	95	20
RREFam012	**Lanthipeptide_RRE_2**	89	20
RREFam013	**LAP_RRE_1**	68	23
RREFam014	LAP_RRE_2	31	25
RREFam015	**Lassopeptide_RRE_1**	97	19
RREFam016	Lassopeptide_RRE_2	94	19
RREFam017	**MNIO_Partner_RRE_1****	95	19
RREFam018	**MNIO_Partner_RRE_2****	98	19
RREFam019	**MNIO_Partner_RRE_3****	89	19
RREFam020	Mycofactocin_RRE	81	25
RREFam021	Pantocin_Microcin_RRE	83	20
RREFam022	Pearlin_RRE_1	84	20
RREFam023	**Pearlin_RRE_2**	58	45
RREFam024	Pearlin_RRE_3	17	20
RREFam025	**Pearlin_RRE_4**	57	30
RREFam026	**PQQ_RRE**	99	19
RREFam027	**Proteusin_RRE_1**	76	25
RREFam028	**Proteusin_RRE_2**	96	19
RREFam029	**Proteusin_RRE_3**	83	20
RREFam030	Proteusin_RRE_4	41	23
RREFam031	Proteusin_RRE_5	93	19
RREFam032	QHNDH_RRE	77	20
RREFam033	Ranthipeptide_RRE_1	95	23
RREFam034	**Ranthipeptide_RRE_2**	95	22
RREFam035	Sactipeptide_RRE_1	88	20

RREFam036	Sactipeptide_RRE_2	84	19
RREFam037	Sactipeptide_RRE_3	91	19
RREFam038	**Sactipeptide_RRE_4**	92	19
RREFam039	**Streptamidine_RRE**	69	30
RREFam040	**Thiopeptide_RRE**	84	20
RREFam041	Trifolitoxin_RRE	18	23
RREFam042	**Cupin_JmjC_RRE**	60	20
RREFam043	**Glutathione_S_Transferase_RRE**	97	19
RREFam044	**Memo_RRE**	72	20
RREFam045	**Metallo_Beta_Lactamase_RRE**	92	19
RREFam046	**Methyltransferas_RRE**	98	19
RREFam047	**Nucleotidyltransferase_RRE**	78	20
RREFam048	**Phosphoribosyl_Transferase_RRE**	99	19
RREFam049	**Foldseek_RRE_1**	66	23
RREFam050	**Foldseek_RRE_2**	93	19
RREFam051	**Foldseek_RRE_3**	97	19
RREFam052	**Foldseek_RRE_4**	62	24
RREFam053	**Foldseek_RRE_5**	60	21
RREFam054	**Foldseek_RRE_6**	72	20
RREFam055	**Foldseek_RRE_7**	46	22
RREFam056	**Foldseek_RRE_8**	63	20
RREFam057	**Foldseek_RRE_9**	93	19
RREFam058	**Foldseek_RRE_10**	86	21
RREFam059	**Foldseek_RRE_11**	85	20

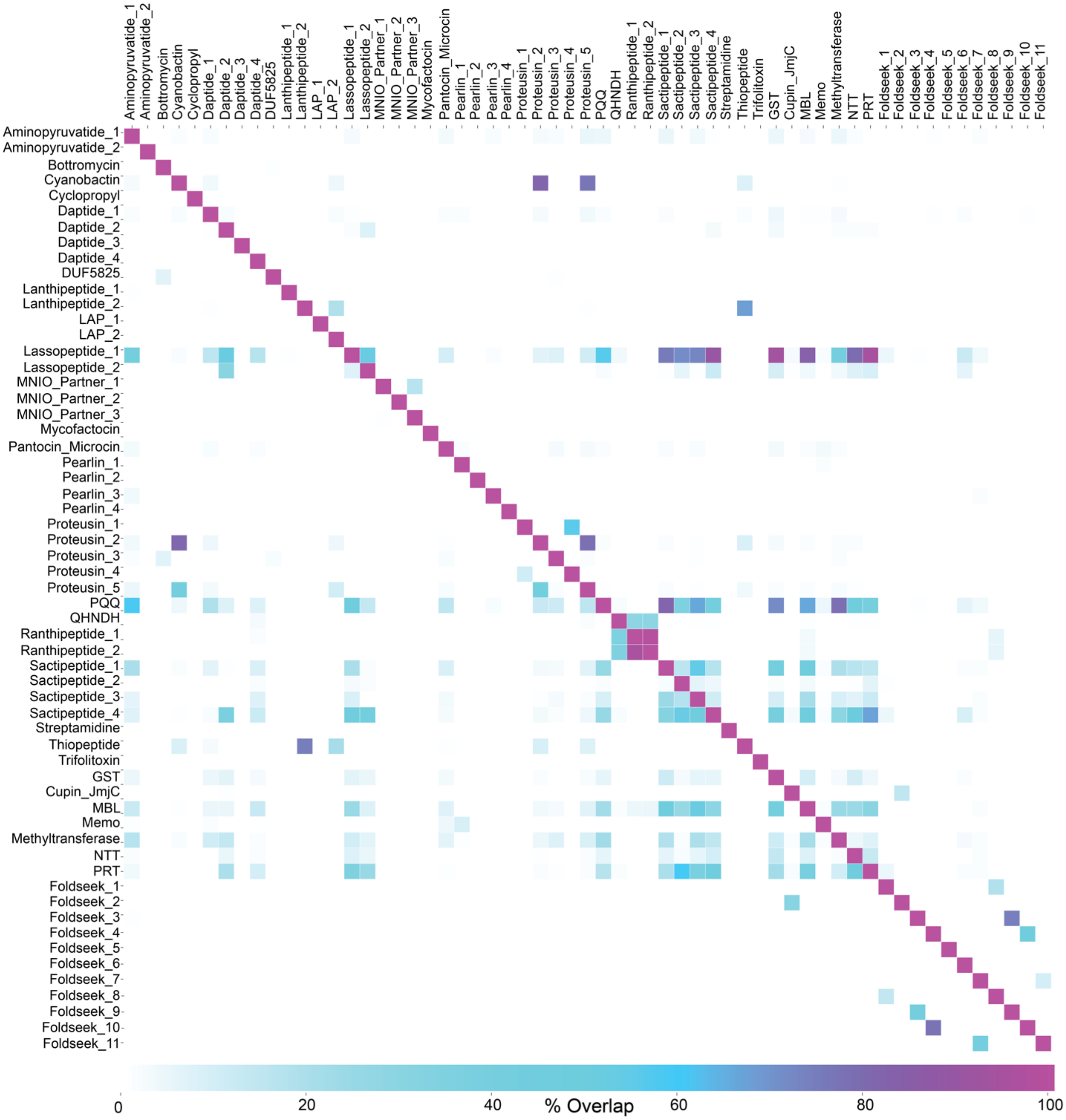

Figure S4: Matrix showing the overlap of all proteins scoring at or above the recommended bitscore cutoffs after filtering. See Table S1 for HMMs in precision mode and cutoff scores.

A

Domain	Total Entries in UniProt	Domain % of UniProt
Archaea	6,604,069	3
Bacteria	160,102,368	64
Eukaryota	75,190,826	30
Other	7,854,628	3

B

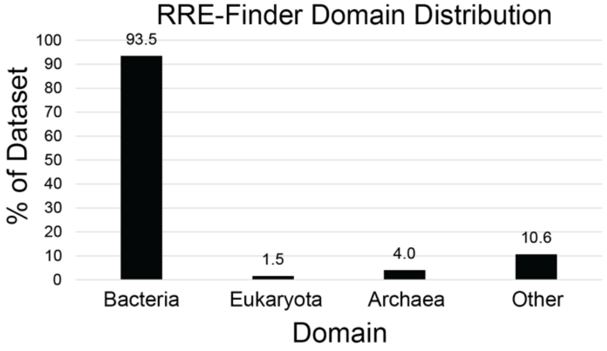

C

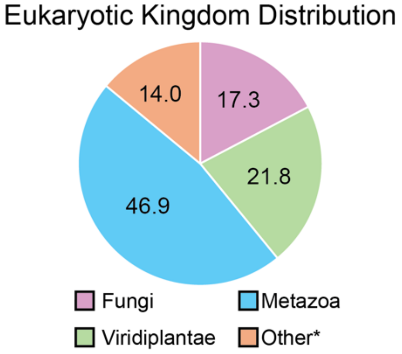

D *Astyanax mexicanus*
A0A3B1JZV8

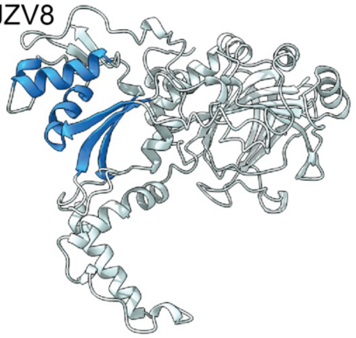

E

Homologous protein ID	Organism	% Identity
A0A4W4FM24	*Electrophorus electricus*	80
A0AAD8ZLJ9	*Electrophorus voltai*	80
A0A7J6AHB9	*Ameiurus melas*	75
A0A8T0B575	*Silurus meridionalis*	75
A0AA88LSY8	*Tachysurus vachellii*	75

F WP_223048796.1
Leptothermofonsia sichuanesis E412

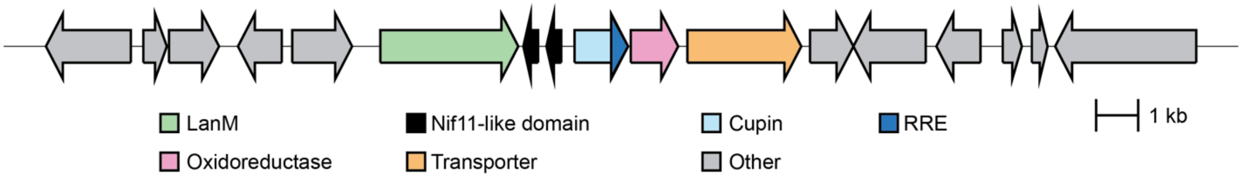

Figure S5: Investigation of putative eukaryotic RRE-containing proteins. A) Taxonomic domain distribution in UniProtKB at the time of dataset collection (Jan 2024). "Others" includes viral proteins lacking UniProt domain annotations. B) Domain distribution of proteins in the RRE-Finder dataset based on UniProtKB annotations. C) Kingdom-level distribution of eukaryotic proteins identified by RRE-Finder according to UniProtKB. “Other*” refers to proteins annotated as NA-Eukaryota in UniProt. D-E) Example metazoan protein with an RRE-like predicted structure (full structure pTM = 0.87) and its closest structural homologs identified in NCBI. F) BGC diagram of a bacterial homolog (32% sequence identity to the eukaryotic RRE-containing protein) with RiPP-like architecture.

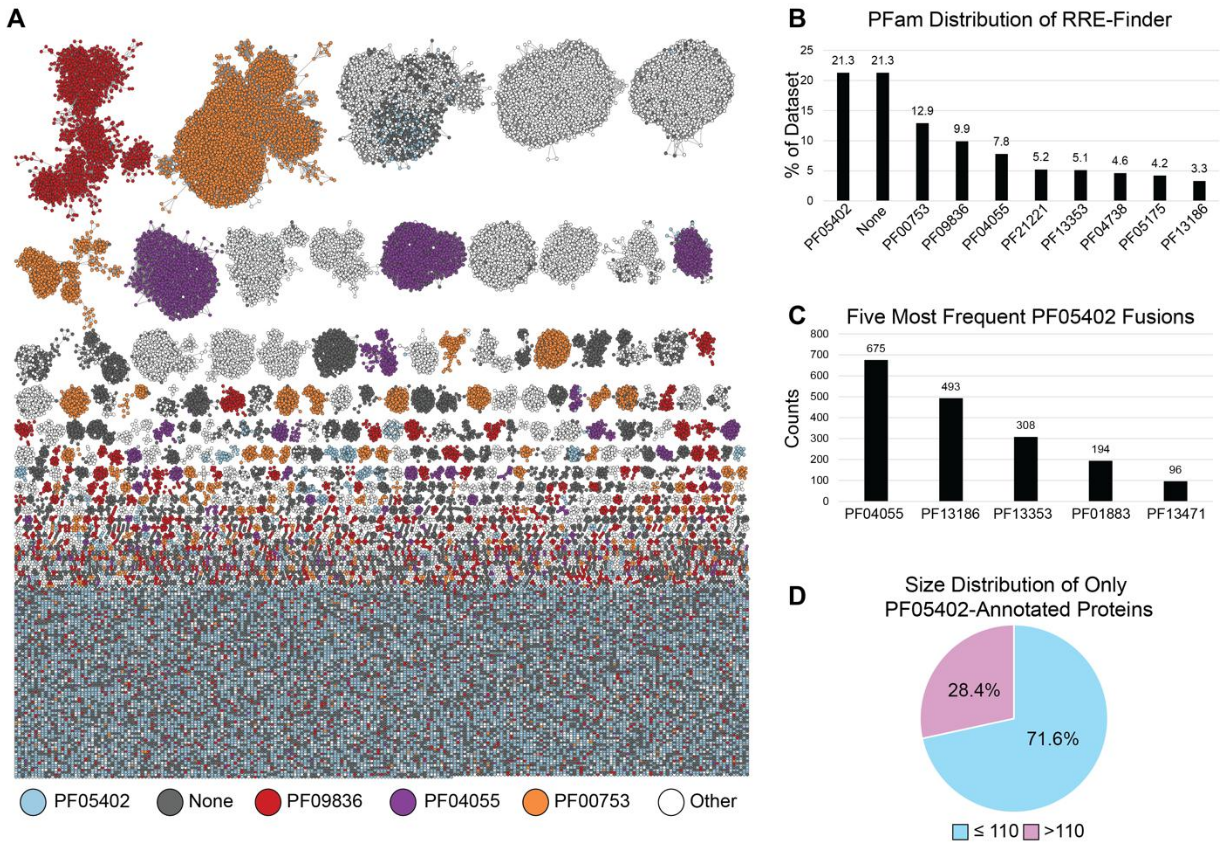

Figure S6: Breakdown of RRE-Finder Pfam distribution and PF05402 analysis. A) Sequence similarity network (alignment score = 60, 91,283 nodes, RepNode = 60) of full-length UniProt sequences showing the most frequent Pfam annotations across the entire RRE-Finder dataset. B) Percentage representation of the 10 most common Pfam annotations within the RRE-Finder dataset. C) Five most frequent additional Pfam annotations found in proteins also annotated with PF05402. D) Size distribution of proteins annotated solely as PF05402, where sequences ≤110 amino acids correspond to discrete RRE domains. All Pfams annotations are as follows: metallo-beta-lactamase superfamily, (PF00753), iron-sulfur cluster assembly protein (PF01883), radical SAM superfamily (PF04055), lantibiotic dehydratase N-terminus (PF04738), methyltransferase small domain (PF05175), PqqD (PF05402), putative DNA-binding domain – now known as MNIO partner protein DUF2063 (PF09836), iron-sulfur cluster-binding domain (PF13186), 4Fe-4S single cluster domain (PF13353), transglutaminase-like superfamily (PF13471), metallo-beta-lactamase-like C-terminal domain (PF21221).

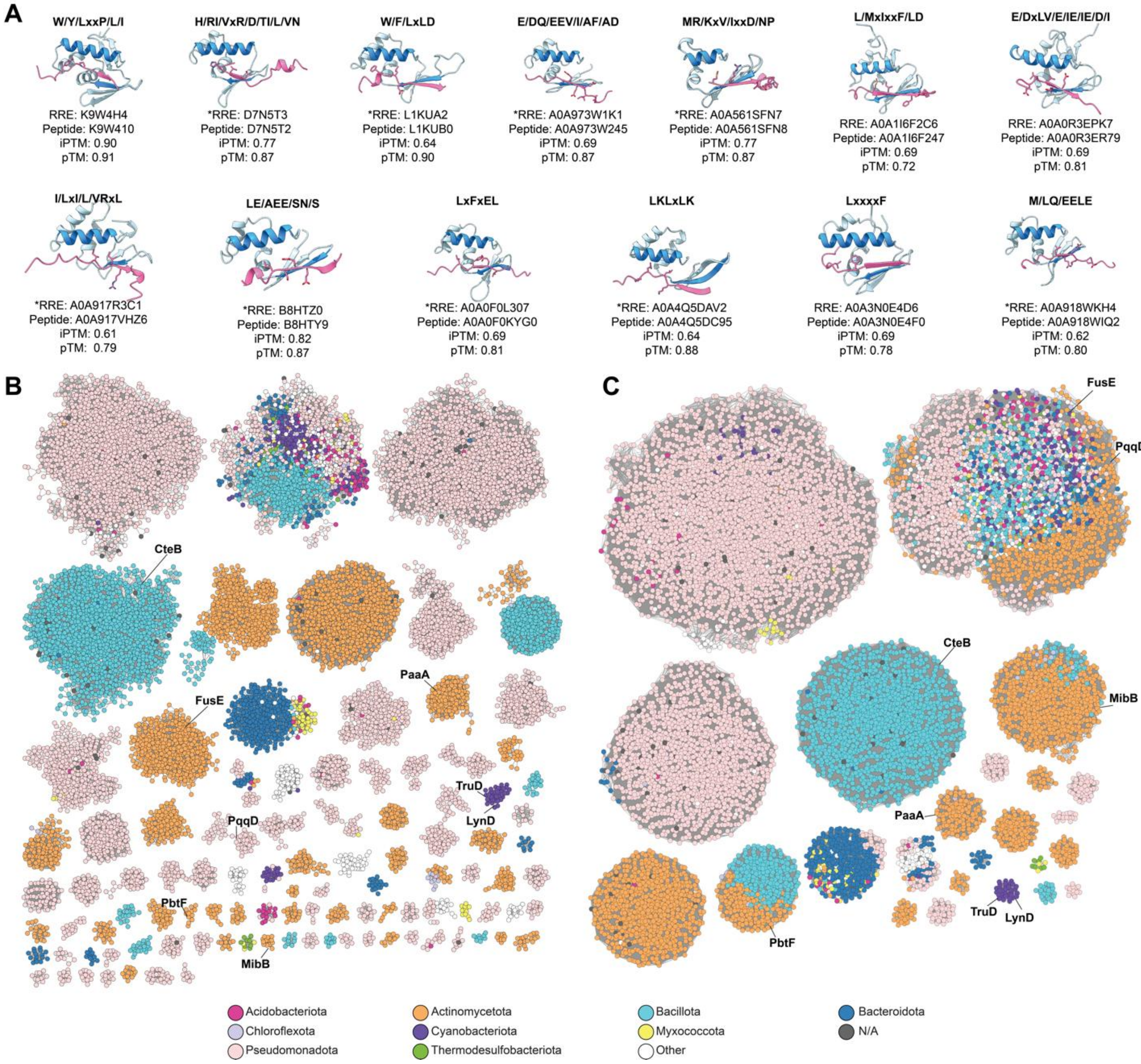

Figure S7: AlphaFold 3-predicted RRE–peptide complex structures representing distinct recognition sequence groups and phylogenic distribution. A) RRE domains are shown in blue, with predicted precursor peptides highlighted in pink. Conserved recognition sequences identified through sequence logos are depicted as sticks (interface predicted Template Modeling score (ipTM) and pTM scores are indicated for each structure). An asterisk (*) denotes cases where the full protein, rather than the excised RRE domain, was used for prediction to improve model confidence, except where the RRE was discrete from the start. B) Recognition Sequence Mapping SSN (Fig. 3) recolored to represent phylum with 44,350 sequences represented as 11,118 nodes (RepNode = 90) and visualized at an alignment score of 20. The ten most populous phyla in the dataset are indicated by color. Examples of structurally characterized RRE domains are annotated. C) Same as panel B but visualized at an alignment score of 10.

A

Sample	$PbtF_{RRE}$ + $PbtA_{13mer}$ (NLNDLPMDVFEMA)
**Data collection**	
Wavelength (Å)	0.97872
Space group	$P3_221$
Cell dimensions	
a, b, c (Å)	40.3, 40.3, 101.8
α, β, γ (°)	90.0, 90.0, 120.0
Resolution (Å)[1]	32.99–1.23 (1.27–1.23)
R_{merge}[1]	0.012 (0.232)
R_{pim}[1]	0.012 (0.232)
$I / \sigma I$[1]	24.06 (2.75)
$CC_{1/2}$[1]	1.000 (0.881)
Completeness (%)[1]	99.76 (100.00)
Redundancy[1]	2.0 (2.0)
**Refinement**	
Resolution (Å)[1]	32.99–1.23 (1.27–1.23)
No. reflections[1]	28,567 (2,805)
R_{work} / R_{free}[1,2]	0.22 (0.27) / 0.26 (0.32)
No. atoms	820
Protein	715
Ligand/ion	1
Water	104
B-factors ($Å^2$)	
Average	19.55
Protein	18.10
Ligand/ion	30.00
Water	29.38
R.m.s. deviations	
Bond lengths (Å)	0.006
Bond angles (°)	0.90

B

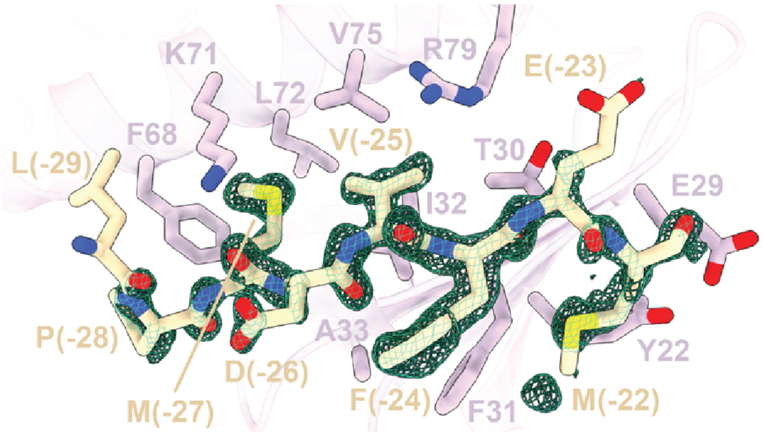

Figure S8: Crystallographic information and interactions between the RRE of PbtF (PbtFRRE) and its leader peptide. A) Table of Crystallographic data collection and refinement statistics. 1. Highest resolution shell is shown in parentheses. 2. $R_{work} = \Sigma(|F_{obs}|-k|F_{calc}|)/\Sigma|F_{obs}|$ and R_{free} is the R value for a test set of reflections consisting of a random 5% of the diffraction data not used in refinement. B) The co-crystal structure between $PbtF_{RRE}$ (purple) and a 13-residue fragment of its leader peptide ($PbtA_{13mer}$, beige) was determined at 1.23 Å (PDB: 8T19), revealing interactions at the RRE/leader peptide interface. An eight-residue fragment of $PbtA_{13mer}$, spanning Leu(-29) to Met(-22), was visible in the difference Fourier (F_{obs}–F_{calc}) electron density map, calculated from the final refined coordinates minus those for the leader peptide, and contoured at 3 σ above background.

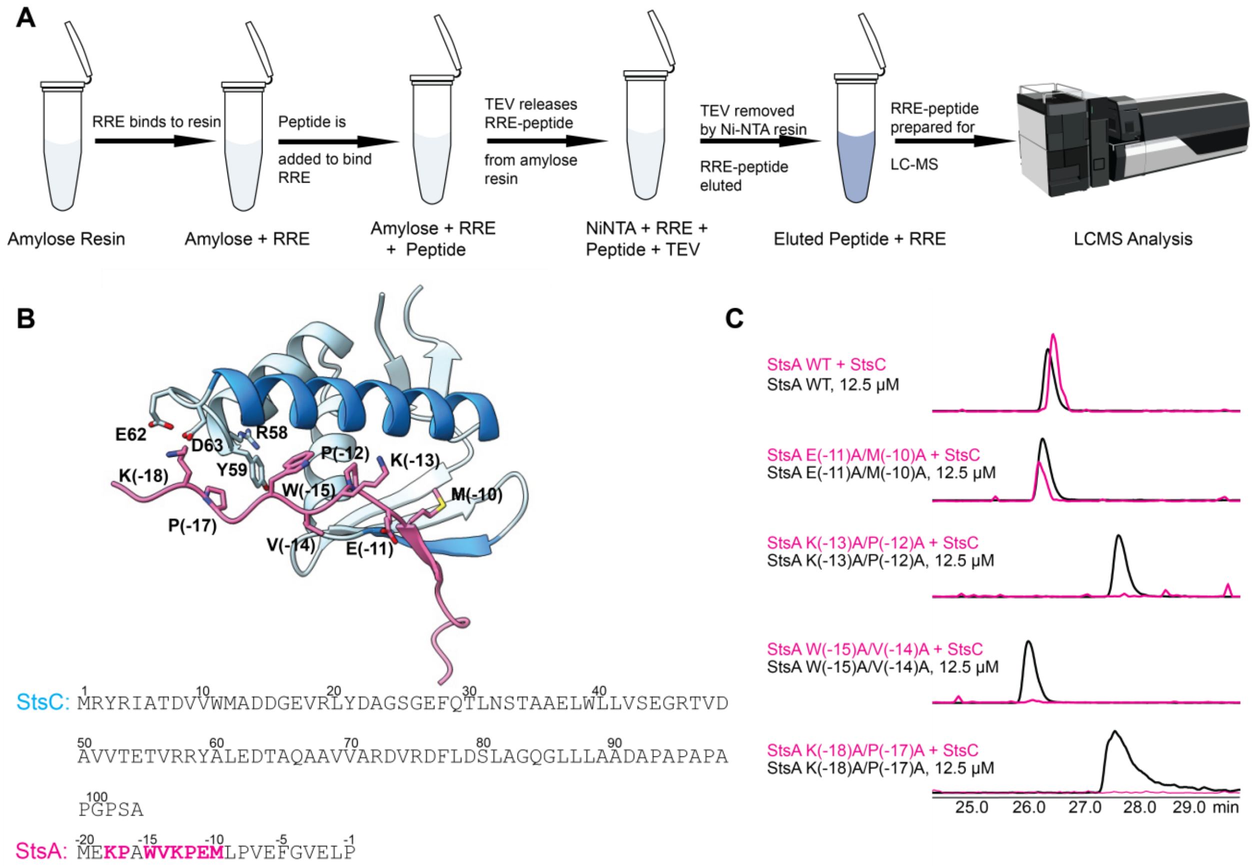

Figure S9: StsA-StsC leader peptide capture assay and results. A) Workflow overview of the pull-down assay involving resin-based binding followed by LC–MS analysis. B) AlphaFold3-predicted complex between StsC (UniProt: A0A1R1S990) and the StsA leader region (residues –20 to –2; NCBI: WP_158080382.1), with varied residues shown as sticks (ipTM: 0.74, pTM: 0.78). C) LC–MS assay results comparing peptide capture of StsC with different StsA double alanine variants. Black traces show peptide standards for charge state and retention time validation. Pink traces display captured peptides for wild-type and variants, scaled to the same y-axis. Extracted ion chromatogram (EIC) traces are shown for $[M + 2H]^{+2}$ ± 0.2 Da with the following masses: StsA WT (residues -20 to -1): 1278.65 Da, StsA E(-11)A/M(-10)A: 1219.75 Da, StsA K(-13)A/P(-12)A: 1237.25 Da, StsA W(-15)A/V(-14)A: 1207.25 Da, StsA K(-18)A/P(-17)A: 1237.20 Da.
